# HMGB1 Mediates Endogenous TLR2 Activation and Brain Tumor Regression

**DOI:** 10.1371/journal.pmed.1000010

**Published:** 2009-01-13

**Authors:** James F Curtin, Naiyou Liu, Marianela Candolfi, Weidong Xiong, Hikmat Assi, Kader Yagiz, Matthew R Edwards, Kathrin S Michelsen, Kurt M Kroeger, Chunyan Liu, A. K. M. Ghulam Muhammad, Mary C Clark, Moshe Arditi, Begonya Comin-Anduix, Antoni Ribas, Pedro R Lowenstein, Maria G Castro

**Affiliations:** 1 Board of Governors' Gene Therapeutics Research Institute and Department of Medicine, Cedars Sinai Medical Center, Los Angeles, California, United States of America; 2 Department of Molecular and Medical Pharmacology, University of California, Los Angeles, California, United States of America; 3 Inflammatory Bowel Disease Center and Immunobiology Research Institute, Cedars Sinai Medical Center, Los Angeles, California, United States of America; 4 Division of Pediatric Infectious Diseases, Cedars Sinai Medical Center, Los Angeles, California, United States of America; 5 Department of Surgery, University of California, Los Angeles, California, United States of America; 6 Jonsson Comprehensive Cancer Center, David Geffen School of Medicine, University of California, Los Angeles, California, United States of America; 7 The Brain Research Institute, University of California, Los Angeles, California, United States of America; Brain Tumor Institute, Cleveland Clinic Foundation, United States of America

## Abstract

**Background:**

Glioblastoma multiforme (GBM) is the most aggressive primary brain tumor that carries a 5-y survival rate of 5%. Attempts at eliciting a clinically relevant anti-GBM immune response in brain tumor patients have met with limited success, which is due to brain immune privilege, tumor immune evasion, and a paucity of dendritic cells (DCs) within the central nervous system. Herein we uncovered a novel pathway for the activation of an effective anti-GBM immune response mediated by high-mobility-group box 1 (HMGB1), an alarmin protein released from dying tumor cells, which acts as an endogenous ligand for Toll-like receptor 2 (TLR2) signaling on bone marrow-derived GBM-infiltrating DCs.

**Methods and Findings:**

Using a combined immunotherapy/conditional cytotoxic approach that utilizes adenoviral vectors (Ad) expressing Fms-like tyrosine kinase 3 ligand (Flt3L) and thymidine kinase (TK) delivered into the tumor mass, we demonstrated that CD4^+^ and CD8^+^ T cells were required for tumor regression and immunological memory. Increased numbers of bone marrow-derived, tumor-infiltrating myeloid DCs (mDCs) were observed in response to the therapy. Infiltration of mDCs into the GBM, clonal expansion of antitumor T cells, and induction of an effective anti-GBM immune response were TLR2 dependent. We then proceeded to identify the endogenous ligand responsible for TLR2 signaling on tumor-infiltrating mDCs. We demonstrated that HMGB1 was released from dying tumor cells, in response to Ad-TK (+ gancyclovir [GCV]) treatment. Increased levels of HMGB1 were also detected in the serum of tumor-bearing Ad-Flt3L/Ad-TK (+GCV)-treated mice. Specific activation of TLR2 signaling was induced by supernatants from Ad-TK (+GCV)-treated GBM cells; this activation was blocked by glycyrrhizin (a specific HMGB1 inhibitor) or with antibodies to HMGB1. HMGB1 was also released from melanoma, small cell lung carcinoma, and glioma cells treated with radiation or temozolomide. Administration of either glycyrrhizin or anti-HMGB1 immunoglobulins to tumor-bearing Ad-Flt3L and Ad-TK treated mice, abolished therapeutic efficacy, highlighting the critical role played by HMGB1-mediated TLR2 signaling to elicit tumor regression. Therapeutic efficacy of Ad-Flt3L and Ad-TK (+GCV) treatment was demonstrated in a second glioma model and in an intracranial melanoma model with concomitant increases in the levels of circulating HMGB1.

**Conclusions:**

Our data provide evidence for the molecular and cellular mechanisms that support the rationale for the clinical implementation of antibrain cancer immunotherapies in combination with tumor killing approaches in order to elicit effective antitumor immune responses, and thus, will impact clinical neuro-oncology practice.

## Abstract 2

Maria Castro and colleagues use cell line and transgenic mouse approaches to study the mechanisms underlying the immune response to glioblastoma multiforme.

## Editors' Summary

**Background.:**

Every year, more than 175,000 people develop a primary brain tumor (a cancer that starts in the brain rather than spreading in from elsewhere). Like all cancers, brain tumors develop when a cell acquires genetic changes that allow it to grow uncontrollably and that change other aspects of its behavior, including the proteins it makes. There are many different types of cells in the brain and, as a result, there are many different types of brain tumors. However, one in five primary brain tumors is glioblastoma multiforme (GBM; also known as grade 4 astrocytoma), a particularly aggressive cancer. With GBM, the average time from diagnosis to death is one year and only one person in 20 survives for five years after a diagnosis of GBM. Symptoms of GBM include headaches, seizures, and changes in memory, mood, or mental capacity. Treatments for GBM, which include surgery, radiotherapy, and chemotherapy, do not “cure” the tumor but they can ease these symptoms.

**Why Was This Study Done?:**

Better treatments for GBM are badly needed, and one avenue that is being explored is immunotherapy—a treatment in which the immune system is used to fight the cancer. Because many tumors make unusual proteins, the immune system can sometimes be encouraged to recognize tumor cells as foreign invaders and kill them. Unfortunately, attempts to induce a clinically useful anti-GBM immune response have been unsuccessful, partly because the brain contains very few dendritic cells, a type of immune system cell that kick-starts effective immune responses by presenting foreign proteins to other immune system cells. Another barrier to immunotherapy for GBM is immune evasion by the tumor. Many tumors develop ways to avoid the immune response as they grow. For example, they sometimes reduce the expression of proteins that the immune system might recognize as foreign. In this study, the researchers test a new combined treatment strategy for GBM in which dendritic cells are encouraged to enter the brain and tumor cells are killed to release proteins capable of stimulating an effective antitumor immune response.

**What Did the Researchers Do and Find?:**

The researchers first established brain tumors in mice. Then, they injected harmless viruses carrying the genes for Fms-like tyrosine kinase 3 ligand (Ftl3L; a protein that attracts dendritic cells) and for thymidine kinase (TK; cells expressing TK are killed by a drug called gancyclovir) into the tumor. Expression of both Flt3L and TK (but not of either protein alone) plus gancyclovir treatment shrank the tumors and greatly improved the survival of the mice. The researchers show that their strategy increased the migration of dendritic cells into the tumor provided they expressed an immune system protein called Toll-like receptor 2 (TLR2). TLR2 expression on the dendritic cells was also needed for an effective anti-tumor immune response and for tumor regression. TLR2 normally activates dendritic cells by binding to specific proteins on invading pathogens, so what was TLR2 binding to in the mouse tumors? The researchers reveal that TLR2 was responding to high-mobility-group box 1 (HMGB1), a protein released by the dying tumor cells by showing that treatment of the tumor-bearing mice with the HMGB1 inhibitor glycyrrhizin blocked the therapeutic effect of Flt3L/TK expression. Finally, the researchers report that other tumor cell types release HMGB1 when they are killed and that the Flt3L/TK expression strategy can also kill other tumors growing in mouse brains.

**What Do These Findings Mean?:**

Results obtained in mouse models of human diseases do not always lead to effective treatments for human patients. Nevertheless, the findings of this study provide new insights into how an effective immune response against brain tumors might be brought about. Most importantly, they show that an effective strategy might need to both attract dendritic cells into the brain tumor and to kill tumor cells, so they release proteins that can activate the dendritic cells. That is, the authors suggest it's important to combine immunotherapies with tumor-killing strategies to provide effective treatments for primary and metastatic brain tumors

**Additional Information.:**

Please access these Web sites via the online version of this summary at http://dx.doi.org/10.1371/journal.pmed.1000010.

- The US National Cancer Institute provides information about brain tumors for patients and health professionals and about the the immune system and how it can be harnessed to fight cancer (in English and Spanish)
- Cancer Research UK provides information on all aspects of brain tumors for patients and their caregivers
- MedlinePlus provides links to further information about brain cancer, (including some links to information in Spanish)
- The American Brain Tumor Association provides brain tumor resources and information
- The National Brain Tumor Society provides educational and support services regarding brain tumors

## Introduction

The systemic immune system can be ignorant of antigens derived from tumors growing within the confines of the brain parenchyma [1] owing to the paucity of professional antigen presenting cells (APCs) and lymphatic drainage. Added to the brain's immune privilege [2], and tumor immune escape [3], these factors pose clinically significant obstacles for successful immunotherapy. The most aggressive intracranial primary brain tumor is glioblastoma multiforme (GBM), which is associated with 5% survival rates 5-y postdiagnosis, despite advances in surgery, chemotherapy, and radiotherapy [4]. Novel experimental therapies that harness the power of the patient's immune system are being pursued to improve therapeutic efficacy [5,6]. We postulated that the lack of dendritic cells (DCs) within the brain parenchyma is central to the failure of glioma cell killing approaches to stimulate a strong adaptive antiglioma immune response. To this effect, we developed a combined gene therapeutic approach aimed at engineering the tumor microenvironment to induce the migration into the tumor mass of APCs, combined with tumor cell death to release tumor antigens. Our approach consists of expressing Fms-like tyrosine kinase 3 ligand (Flt3L), which induces DC infiltration into the brain parenchyma [7], in combination with the conditional cytotoxic gene thymidine kinase (TK) [8].

Cell death can trigger antigen-specific immune responses by releasing tumor antigens and signals that mediate phagocytosis by DCs and also DC maturation. Tumor cell death caused by radiation can induce DC-mediated cytotoxic T-lymphocyte (CTL) responses and long-term antitumor immunity [9]. These events can be triggered by the release of endogenous Toll-like receptor (TLR) ligands from dying cells [9] and can lead to either tolerance or auto-immunity [10,11]. TLR signaling induces production of pro-inflammatory cytokines and upregulation of costimulatory molecules, which result in the activation of the adaptive immune system [12]. In this context, it has recently been shown that TLR2 can sense pancreatic β-cell death and contribute to the initiation of auto-immune diabetes [13].

The development of many types of cancers has been linked to chronic inflammation [14]. Inflammation is linked to increased risks of tumorigenesis through alterations in tumor cell survival, proliferation, and metastasis; modulation of adaptive immune responses; increases in tumor angiogenesis; and modifying the tumor microenvironment, thus affecting the efficacy of antitumor therapies. Pro-inflammatory cytokines (e.g., tumor necrosis factor-α [TNFα] and interleukin-6 [IL-6] secreted by macrophages and other innate immune cells), as well as downstream signaling pathways (e.g., RAS and myc) and the activities of transcription factors such as NF-_κ_B and STAT3, have all been linked to malignant transformation, and are also involved in tumor-related inflammation [14–19]. While TLRs play an important role in detecting and regulating the innate immune response to bacteria, viruses, and/or parasites, TLRs are also necessary for the induction of an adaptive immune response through the activation and maturation of DCs and macrophages [20,21]. Herein we demonstrate that codelivery of TK (+ systemic gancyclovir [GCV]) to kill glioma cells and release putative endogenous TLR ligands, with Flt3L to attract DCs to the brain, results in a powerful, CD8+ T cell-mediated systemic immune response that elicits tumor regression and immunological memory. TLR2 expression on bone marrow-derived DCs (BMDC) was necessary to stimulate a systemic antiglioma immune response. Activation of TLR2 signaling was essential for two key stages in priming immune responses against brain tumor antigen: (1) the migration of peripheral DC into the brain tumor, and (2) the subsequent activation of DC and stimulation of tumor antigen specific T cell clonal expansion.

We identified high-mobility-group box 1 (HMGB1) as an endogenous TLR2 agonist that was released from dying tumor cells, both in vitro and in vivo in response to several tumor cell killing approaches, i.e., adenoviral vector (Ad)-TK (+GCV), radiation, and temozolomide. Blocking HMGB1 activity in vivo using glycyrrhizin or specific anti-HMGB1 neutralizing antibodies, inhibited Flt3L/TK-induced brain tumor regression. Thus, tumor-derived HMGB1 elicits endogenous TLR2 signaling and initiates a CD8^+^ T cell-dependent anti-GBM immune response.

The clinical impact of these data is highlighted by the recent description of subpopulations of human cancer patients containing SNPs or microsatellite polymorphisms in TLR2, or the heterodimeric receptors TLR1 and TLR6 [22–26]. Patients that harbor mutations in the TLR2 signaling pathway have significantly poorer clinical outcome and more rapid progression of gastric, colorectal, lymphoma, and prostate cancer than patients with normal TLR2 signaling components [22–26]. It is thus likely that our conclusions related to antiglioma immune responses may be extended to enhancing immune responses against other cancers. Further, our data also provide support to the implementation of antibrain cancer immunotherapies in combination with tumor killing approaches in the clinical setting.

## Methods

### Ads, Cell Lines, Plasmids, and Reagents

We used first generation, recombinant Ads (serotype 5) expressing Herpes Simplex Virus Type I-TK (Ad-TK) [8,27], Flt3L (Ad-Flt3L) [7], and an Ad vector with no transgene (Ad0) in this study [8]. The methods for adenoviral generation, purification, characterization, and scale-up have been previously described by our lab [28]. GL26 and GL261 were obtained from NCI; LLc1 and B16-F10 cells were obtained from ATCC; and HEK-293 cells were obtained from Microbix. CNS1 were a gift from W. Hickey (Department of Pathology, Dartmouth Medical Center, Lebanon, New Hampshire, USA) and DSL6A were a gift from D. Longnecker (Department of Pathology, Dartmouth Medical Center). HEK293 cells were cultured in MEM supplemented with nonessential amino acids, 1% Pen-Strep, 1% L-Glutamine (all from CellGro), and 10% FCS (Omega Scientific). All other cell lines were grown in DMEM culture media (CellGro) supplemented with 10% FCS and 1% Pen-Strep and passaged routinely every 2–3 d. pCMV-TLR2 and pCMV-TLR4 were a gift from Mike Roth [29] and pEF1α-MD2 was a gift from Kensuke Miyake [30]. pGL3-NFκB and pRL-TK were purchased from Promega. PAM3CSK4 and LPS were purchased from Invivogen. Dual Luciferase assay was purchased from Promega. IL6 and TNFα ELISA kits were purchased from Ebioscience. The fluorescent-conjugated immunoglobulins CD3-PE, CD3-PerCP, CD4-PerCP, CD8a-FITC, CD8a-PE, CD11c-PE, CD45-PE, CD45-PerCP, and MHC II-FITC were all purchased from BD Biosciences. r-PE labeled Trp2_180–188_ loaded MHC I tetramers were obtained from Beckman Coulter Inc. and recombinant human IL-2 (Proleukin) was purchased from Novartis Pharmaceuticals Corporation. The LPS detection kit QCL-1000 Chromogenic LAL Endpoint assay was purchased from Lonza and EndoFree Plasmid Maxi kit was purchased from Qiagen. Temozolomide (Temodar) was obtained from Schering Corporation and provided by the CSMC Pharmacy.

### Transgenic Mouse Models and Mixed Chimeric Mice

GL26 cells did not contain Y chromosomal sequences so were derived from a primary tumor in a female C57BL/6 mouse (Figure S1). Consequently, female C57BL/6 mice were used throughout this study. *MyD88^−/−^* and *TLR4^−/−^* mice have been previously described [31] and were kindly provided by S. Akira (Osaka University, Osaka, Japan). We backcrossed *MyD88^−/−^* for ten generations and *TLR4^−/−^* for six generations into the C57BL/6J strain to generate a homogenous population. Wild-type (WT) C57BL/6J, Balb(c), *TLR2^−/−^* (B6.129-*Tlr2^tm1Kir^*/J stock number 004650)*, CD4^−/−^* (B6.129S2-*Cd4^tm1Mak^*/J stock number 002663), *CD8^−/−^* (B6.129S2-*Cd8a^tm1Mak^*/J stock number 002665), *Rag1^−/−^* (B6.129S7-*Rag1^tm1Mom^*/J stock number 002216), and green fluorescent protein (*GFP)^+/+^* (C57BL/6-Tg(UBC-GFP)30Scha/J stock number 004353) mice, all on a C57BL/6J background were purchased from Jackson Laboratories. Two sets of chimeric mice were used in this study. We generated chimeric mice using WT C57BL/6 mice and *GFP^+/+^* mice (Figure S2) to assess the origin of tumor-infiltrating DC. We also made chimeric mice using WT C57BL/6 mice and *TLR2^−/−^* mice to assess the role of TLR2 signaling on bone marrow- and microglia-derived cells. To establish mixed chimeric mice, we first irradiated recipient mice (7 Gy) using an irradiator with a ^137^Cs source and allowed the mice to recover for 4 h in their home cages. We humanely killed adult donor mice (4–6 wk old by CO_2_ asphyxia and cervical dislocation) and extracted bone marrow from the long bones. Recipient mice were anaesthetized the same day with an IP injection of ketamine (75 mg/kg) and medetomidine (0.5 mg/kg), and placed under a heat lamp for 5 min. 10^7^ bone marrow cells in 100 μl were then transferred via tail vein injection into recipient irradiated mice using a tuberculin syringe with a 31-gauge needle. Atipamazole (IP) was used to recover mice from anesthesia. Bone marrow in the recipients was allowed to reconstitute for 10 wk prior to tumor implantation and subsequent treatment. We established four chimeric mouse lines: WT mice with *TLR2^−/−^* bone marrow; *TLR2^−/−^* mice with WT bone marrow; WT mice with *GFP^+^* bone marrow; and *GFP^+^* mice with WT bone marrow. Generally, repopulation of irradiated mice is not 100% efficient [32], and GFP expression is not uniformly expressed in all cells [33], therefore data need to be compared and normalized to the percentage of green immune cells present in normal GFP^+/+^ mice.

### Glioma Model

To establish a syngeneic intracranial brain tumor model, we used GL26 cells [34,35] derived from a female C57BL/6 mouse intracranial neoplasm (Figure S1). Other intracranial brain tumor models tested in C57Bl/6 mice were: GL261 cells (glioma) or B16-F10 cells (melanoma). Female mice (6–12 wk) were anesthetized with an IP injection of ketamine (75 mg/kg) and medetomidine (0.5 mg/kg), placed in a stereotactic apparatus modified for mice, and a hole was drilled in the skull. 2 × 10^4^ GL26, 2 × 10^4^ GL261 cells [36], or 1 × 10^3^ B16-F10 cells [37] in 0.5 μl PBS were injected unilaterally into the right striatum (+0.5 mm AP, +2.2 mm ML, −3.0 mm DV from bregma) using a 5-μl Hamilton syringe with a 33-gauge needle. The needle was left in place for 3 min prior to removal to allow tumor cells to settle at the injection site. Mice were resuscitated using atipamazole (IP) and administered buprinex (SQ) as an analgesic. 17 d after tumor implantation and using the same stereotactic coordinates, mice received an intratumoral injection of either 1 × 10^8^ IU Ad0, 5 × 10^7^ IU Ad-Flt3L + 5 × 10^7^ IU Ad0, 5 × 10^7^ IU Ad-TK + 5 × 10^7^ IU Ad0, or 5 × 10^7^ IU Ad-Flt3L + 5 × 10^7^ IU Ad-TK in 1 μl saline. Alternatively, mice were injected with 1 μl saline as a control. Mice treated with Ad-TK also received 25 μg/kg GCV (IP, Roche) twice daily for 7 d starting the day after vector injection. To perform tumor cell rechallenge into the contralateral brain hemisphere, mice were anaesthetized as described above, a hole was drilled in the skull, and 2 × 10^4^ GL26 cells in 0.5 μl PBS were injected at the following coordinates (+0.5 mm AP, −2.2 mm ML, −3.0 mm DV from bregma). All animal experiments were performed after prior approval by the Institutional Animal Care and Use Committee at Cedars Sinai Medical Center and conformed to the policies and procedures of the Comparative Medicine Department. Mice used in this study were monitored for signs of moribund behavior and euthanized when their health status reached criteria established by the guidelines of the IACUC. Animals were humanely killed by terminal perfusion with oxygenated, heparinized Tyrode's solution (132 mM NaCl, 1.8 mM CaCl_2_, 0.32 mM NaH_2_PO_4_, 5.56 mM glucose, 11.6 mM NaHCO_3_, and 2.68 mM KCl).

### IFNγ ELISPOT

10^6^ splenocytes were purified from tumor-bearing mice (WT C57BL/6 or *TLR2^−/−^*) treated with intratumoral injections of saline or Flt3L and TK. Splenocytes were next prestimulated on a monolayer of fixed GL26 cells (1% PFA for 15 min at RT) in the presence of 10 U/ml IL-2 for 14 d. Fresh IL-2 (at the same concentration) was added every fourth day. Myeloid DCs (mDC) (CD11c+, CD45+, MHC II +) were stained as described in the flow cytometry section and then purified from naïve WT C57BL/6 mouse splenocytes by cell sorting (MoFlow, DAKO). To prepare cellular extracts, 1 × 10^6^ GL26 cells or LLc1 cells were first heat shocked in a 25-cm^2^ tissue culture flask (42 °C, 1 h), allowed to recover (37 °C, 2 h), and then freeze thawed by transferring frozen from liquid N_2_ into 37 °C three times. Cell membranes and other insoluble debris were then removed by centrifugation (12,000 × *g*, 15 min, 4 °C), and protein concentration was determined by BCA assay (Peirce). Purified mDC were loaded with tumor cell extracts by culturing in 2 ml media (in a six-well plate) containing 100 μg/ml GL26 cell extract or 100 μg/ml LLc1 cell extract for 12 h at 37 °C and then were fixed in 1% PFA (in PBS) for 5 min at RT before washing three times with PBS. 1 × 10^5^ stimulator cells (mDC loaded with GL26 or LLc1 cell extract) were incubated with 1 × 10^5^ responder cells (prestimulated T cells) in triplicate for 48 h on 96-well plates coated with mouse anti-IFNγ capture antibody (R&D Systems mouse IFNγ ELISPOT Development kit). IFNγ spots were developed with an anti-mouse IFNγ development antibody as recommended by the manufacturer's detailed instructions (R&D Systems mouse IFNγ ELISPOT Development kit). Concanavalin A (1 μg/ml, 4 h) (Sigma) stimulation of WT and TLR2^−/−^ splenocytes was used as a control in lieu of stimulation with tumor extract pulsed DC.

### Reverse Transcriptase-PCR

RNA was extracted from GL26 cells using RNABee reagent (Tel-test). A reverse transcriptase reaction was set up using 0.5 μg RNA and oligo-dT primer with MMLV Reverse Transcriptase (Promega) (75 °C, 10 min; 42 °C, 1 h; 4 °C, ∞). Primers specific for nucleotides 520 to 951 of mouse GAPDH coding sequence (forward 5′-ACC ACA GTC CAT GCC ATC AC-3′; reverse 5′-TCC ACC ACC CTG TTG CTG TA-3′) (Operon) or nucleotide 676 to 1,176 of mouse Trp2 coding sequence (forward 5′-TGG CTG GAA AGA GAA CTC CA-3′; reverse 5′-GTA AAA GAG TGG AGG ACC ACA-3′) (Operon) were subsequently used to amplify target sequences from the cDNA using PCR (Taq, Promega, 95 °C, 1 min; 55 °C, 1 min; 72 °C, 1 min). DNA bands were separated using agarose gel electrophoresis (IscBioExpress, 1% w/v) and visualized using a gel documentation system (Alpha Innotech Corporation).

### Isolation of Immune Cells

Splenocytes were harvested and red blood cells were removed by incubating in 3 ml ACK solution (0.15 mM NH_4_Cl, 10 mM KHCO_3_, and 0.1 mM sodium EDTA at [pH 7.2]) for 3 min. Splenocytes were then washed in RPMI media (containing 10% FBS, 1% PS, 1% L-Gln). Cervical (draining) lymph nodes (dLNs) were homogenized using a Tenbroeck homogenizer. Tumor-infiltrating immune cells were purified by perfusing tumor-bearing mice with 100 ml (oxygenated) heparinized Tyrode's Solution 7 d after intratumoral injection of Ad-Flt3L and Ad-TK, or Ad0 or saline. Brains were removed from the skull and carefully separated from the meninges with scissors. The tumor was carefully dissected with a scalpel blade and removed avoiding the ventricles. The tumor tissue was diced with a razor blade before homogenizing in RPMI medium (CellGro) using a glass Tenbroeck homogenizer (Kontes). Mononuclear cells were purified from brain tissue by centrifugation (600 × *g*) through a Percol step gradient (70% to 30% Percol in PBS) for 20 min in 15-ml falcon tubes (GE Healthcare); mononuclear cells migrate to the interface between 30% and 70% Percol. The mononuclear cells on the interphase were removed into 10 ml fresh media, washed, and counted using Trypan blue.

### Antibodies, Cell Staining, and Flow Cytometry

Immune cells were labeled with antibodies in cell surface staining buffer (0.1M PBS, w/o Ca^++^, Mg^++^, with 1% FBS, 0.1% sodium azide) for analysis by flow cytometry using a FACScan flow cytometer (Beckton Dickenson). To analyze mDC (CD11c^+^ MHC II^+^ CD45^+^), we stained with CD11c-PE, MHC II-FITC, and CD45-PerCP. To determine the origin of DC in the tumors using GFP chimeric mice, we isolated tumor-infiltrating immune cells as described above and stained with CD11c-PE and CD45-PerCP. Microglia (from the chimeric mice) (CD11b^+^ CD45^int^) were stained with CD11b-PE and CD45-PerCP. GFP+ and GFP− cells were detected on the FL-1 channel of the flow cytometer. Other immune cells analyzed during this study (unpublished data) were macrophages (CD11b^+^ MHC II^+^ CD45^hi^), CD4^+^ T lymphocytes (CD3^+^ CD4^+^ CD8a^−^), CD8a^+^ T lymphocytes (CD3^+^ CD4^−^ CD8a^+^), NK cells (CD3^−^ CD161^+^ CD45^+^), NK-T cells (CD3^+^ CD161^+^ CD45^+^), and B lymphocytes (CD3^−^ CD19^+^ CD45^+^). Antibodies were purchased from BD Biosciences. Mouse anti-HMGB1 was purchased from Sigma and HRP-conjugated antibodies (rabbit anti-mouse and goat anti-rabbit) were purchased from Dako.

### T Cell Proliferation Assay

Splenocytes were purified from tumor-bearing WT C57BL/6 mice and from TLR2^−/−^ mice previously treated with intracranial injections of Ad-Flt3L + Ad-TK or with saline 7 d earlier. Red blood cells were removed using ACK as outlined above. 10^7^ splenocytes were stained with 4 μM CFSE (Invitrogen) for 30 min at RT in complete RPMI media. During cell division, CFSE is equally distributed amongst daughter cells, with each daughter cell inheriting half the original CFSE. Therefore, each daughter cell will only retain half the original fluorescence while cells that do not divide will not lose any fluorescence. Thus, the sequential halving of the mean CFSE fluorescence can determine the number of cell divisions each cell has undergone. Cells were washed with RPMI media and incubated with or without 1 μg/ml Trp2_180–188_ peptide (SVYDFFVWL) for 5 d. Cells were stained with CD3-PE and CD4-PerCP and T cell proliferation was analyzed by flow cytometry. The total number of T cells that had less than one-fourth their original fluorescence (i.e., had divided two or more times) were quantified using flow cytometry and expressed as a percentage of the total T cell population. Data were analyzed using DAKO Summit 4.1.1 software.

### Proliferation of T Cells in Response to IL-2

Tumor-bearing WT (C57BL/6) or TLR2^−/−^ mice were treated 17 d after tumor cell implantation with intratumoral injections saline, or with Ad0, Ad-Flt3L + Ad0, Ad-TK + Ad0, or Ad-Flt3L + Ad-TK as outlined above. T cells were labeled with CD3-PerCP and purified (by cell sorting) from the draining (cervical) LN 7 d after treatment. T cells were then labeled with 4 μM CFSE (Invitrogen) for 30 min (10^7^ cells/ml) at 37 °C before washing twice in 10 ml RPMI media. They were then cultured in 96-well plates (100,000 cells per well) in 200 μl X-VIVO (Cambrex) for 5 d in the presence of 10 U/ml recombinant human IL-2 at 37 °C. T cells were then stained with CD3-PE and the total number of T cells that had less than one-fourth their original fluorescence (i.e., had divided two or more times) were quantified using flow cytometry and expressed as a percentage of the total T cell population.

### Determining the Origin of Tumor-Infiltrating DC

Chimeric mice were used to assess the origin of CD11c^+^ mDC infiltrating into the tumor. We established mixed chimeric mice using the principle that microglial cells are relatively radio resistant whereas bone marrow is radio sensitive. WT and GFP^+/+^ mice were used as controls. Chimeras were as follows: WT mice were irradiated and adoptively transferred with GFP^+/+^ bone marrow; or GFP^+/+^ mice were irradiated and adoptively transferred with WT bone marrow. GL26 cells were implanted in these mice 10 wk after irradiation and treated 17 d later with Ad-Flt3L and Ad-TK. 7 d after the treatment tumor infiltrating immune cells and splenocytes were isolated; flow cytometry was performed to detect total GFP^+^ splenocytes, GFP^+^ tumor-infiltrating microglia, or GFP^+^ tumor-infiltrating mDC. In GFP^+/+^ mice, 83% of splenocytes, 55% of microglia, and 29% of mDC were observed to be GFP^+^. In WT mice 2% of splenocytes, 0.05% of microglia, and 0% of mDC were observed to be GFP^+^. Normalized values of %GFP^+^ immune cells encountered within spleens of the chimeric mice were calculated as follows: Normalized %GFP^+^ immune cells in spleens of WT mice adoptively transferred with GFP^+/+^ bone marrow = (%GFP^+^ splenocytes: 87% − 2%)/(83% − 2%) = 104%. Normalized %GFP^+^ immune cells in spleens of GFP^+/+^ mice adoptively transferred with WT bone marrow = (%GFP^+^ splenocytes: 14% − 2%)/(83% − 2%) = 15%. The equation utilized to normalize the value of %GFP^+^ microglia encountered within tumors of chimeric mice was as follows: Normalized %GFP^+^ microglia infiltrating the tumors of WT mice adoptively transferred with GFP^+/+^ bone marrow = (% intratumoral GFP^+^ microglia in the tumor: 11% − 0.05%)/(55% − 0.05%) = 20%. Normalized %GFP^+^ microglia infiltrating the tumors of GFP^+/+^ mice adoptively transferred with WT bone marrow = (% intratumoral GFP^+^ microglia in the tumor: 53% − 0.05%)/(55% − 0.05%) = 98%. The equation utilized to normalize the value of %GFP^+^ mDC encountered within tumors of the chimeric mice was as follows: Normalized %GFP^+^ mDC infiltrating the tumors of WT mice adoptively transferred with GFP^+/+^ bone marrow = (% intratumoral GFP^+^ mDC in the tumor: 22% − 0%)/(29% − 0%) = 76%. Normalized %GFP^+^ mDC infiltrating the tumors of GFP^+/+^ mice adoptively transferred with WT bone marrow = (% intratumoral GFP^+^ mDC in the tumor: 2% − 0%)/(29% − 0%) = 7%.

### Identifying Whether TLR2 Expression Is Necessary on Bone Marrow- or Microglia-Derived Cells

Two bone marrow chimeric mouse groups were generated using TLR2^−/−^ and WT donors and recipients respectively. Mice were allowed to regenerate their bone marrow for 10 wk before intracranial implantation of GL26 tumor cells as described above. Tumors were allowed to grow for 17 d before treatment with 5 × 10^7^ IU Ad-Flt3L and 5 × 10^7^ IU Ad-TK (+GCV). Mice were monitored daily and euthanized as described above when moribund. A Mantel log-rank test was used to calculate significant differences between groups.

### Culture of DC from Bone Marrow for Subsequent Intratumoral Injection

BMDC were generated as described previously [38] with modifications. Mice were humanely killed by CO_2_ asphyxiation followed by cervical dislocation. Bone marrow was extracted from the long bones and a single cell suspension of bone marrow was created by pipetting up and down with a P1000 pipette. BMDC were cultured from hematopoietic stem cell precursors by supplementing the media with 10 ng/ml Flt3L conditioned media (generated by infecting COS-7 cells with Ad-Flt3L). Media was replaced with 10 ng/ml fresh Flt3L conditioned media every 2–3 d. By day 7, >90% of loosely adherent cells were CD11c^+^ (unpublished data) as determined by flow cytometry. Loosely adherent cells were removed and used for subsequent intratumoral injection and phagocytosis/activation/antigen presentation assays.

### Preparation of Apoptotic and Necrotic Cell Extracts

Necrotic cell extracts were made from GL26 cells (or other tumor cell lines) as outlined in the IFNγ ELISPOT protocol. Cell extracts were used fresh or were frozen at −80 °C for future use. Apoptotic GL26 cells were generated by infecting GL26 cells (in a T25 flask, 0.5 × 10^6^ cells per flask) with Ad-TK (MOI 1,000 IU/cell). After 24 h, media was replaced with fresh media containing 25 μM GCV. A final concentration of 10 μM GCV was added fresh to the cells every day, until the third or fourth day when we detected >80% apoptosis using Annexin V-FITC (Bender MedSystems) and Propidium Iodide (Sigma) staining followed by flow cytometry analysis. Cells that were positive for Annexin V-FITC were quantified to determine the percentage apoptosis.

### Assessment of Apoptosis In Vitro

The pattern of DNA cleavage was analyzed by DNA ladder electrophoresis. Briefly, GL26 cells were infected (MOI 1,000 IU/cell) with Ad-TK (+GCV), Ad-TK, Ad-0, or mock infected. 72 or 96 h postinfection DNA was isolated from 5 × 10^6^ − 1 × 10^7^ cells using an Apoptotic DNA Ladder kit (Roche Diagnostics). DNA was subjected to electrophoresis on 2% agarose gel for 90 min at 45 V. DNA was stained with ethidium bromide.

### Elimination of Brain Tumors with Intratumoral Delivery of BMDC Containing GL26 Cell Extracts

2 × 10^4^ GL26 cells were implanted into the striatum of C57BL/6 mice as outlined before. BMDC were cultured from bone marrow of WT C57BL/6 mice and TLR2^−/−^ mice in IL4 and GM-CSF as described previously. After 7 d, 10^6^ WT and TLR2^−/−^ BMDC were loaded with 100 μg/ml GL26 cell extract in 2 ml RPMI media for 4 h at 37 °C before centrifuging, resuspending at 25,000 cells/μl in PBS, and injecting directly into the 7-d intracranial GL26 brain tumor. Mice were monitored and euthanized as described earlier.

### Tumor Antigen Phagocytosis Assay

GL26 cells were labeled with the fluorescent dye PKH67 (Sigma) as follows; Cells were resuspended at 1 × 10^7^ cells/ml in 1 ml diluent C from the PKH67 Green Fluorescent Cell Linker kit (Sigma) in a 15-ml conical tube before addition of 2 × 10^−6^ M PKH67 dye while vortexing rapidly for 5 s. Cells were incubated for 5 min at RT before adding 2 ml of 1% BSA solution (Sigma) in Dulbecco's PBS (CellGro) to stop any further labeling of cells. 4 ml RPMI complete media was added to the sample and cells were washed four times with 10 ml RPMI complete media before resuspending in 1 ml PBS. Cell extracts were next prepared by rapidly freeze thawing three times as described above. 0.1 mg/ml of PKH67 labeled GL26 cell extract was added to 1 × 10^6^ WT C57BL/6 BMDC or 1 × 10^6^ *TLR2^−/−^* BMDC, previously cultured for 7 d with IL4 (10 ng/ml) and GM-CSF (10 ng/ml). BMDC were incubated 4 °C or at 37 °C for 16 h and washed with 1 ml FACS staining buffer before staining with CD11c-PE and CD45-PerCP as described above and analyzed by flow cytometry using a FACScan flow cytometer (Beckton Dickenson). The time course analysis was performed between 0 to 6 h.

### ELISA

HMGB1 expression was determined in mouse serum using a specific anti-HMGB1 ELISA (Shino Test Corporation) following the manufacturers protocol. Briefly, 100 μl sample diluent was added to each well. Next, 10 μl of mouse serum was added to each well and incubated at 37 °C for 24 h. Wells were washed five times with wash buffer and incubated for 2 h at 25 °C with 100 μl of POD-conjugate solution. Wells were washed a further five times in wash buffer and incubated for 30 min at room temperature with substrate solution. The reaction was stopped by adding 100 μl of stop solution to each well and the absorbance was read at 450 nm (the background was substracted by measuring absorbance at 570 nm). Expression of mouse TNFα and mouse IL6 from BMDC stimulated with PAM3CSK4 or necrotic/apoptotic GL26 cell extracts was determined using ELISA following the manufacturer's protocol exactly (Ebioscience). Briefly, DC previously cultured from bone marrow for 7 d in GM-CSF and IL4 were cultured in 96-well plates at 50,000 cells per well in 200 μl of RPMI + 10% FBS. 0.1 mg/ml GL26 cell extracts or 100 ng/ml PAM3CSK4 were added to BMDC in culture and cells were incubated for 24 h (at 37 °C). Media was removed and 50 μl was used to detect either TNFα or IL6 by ELISA. To do so, 96-well ELISA plates (Corning Costar 9018) were coated with the appropriate capture antibody diluted in coating buffer overnight at 4 °C. The coated plates were washed the next day by aspirating and washing five times with wash buffer (1 × PBS + 0.05% Tween-20), blocked with assay diluent at RT for 1 h, and then washed five times with wash buffer. Samples were diluted 1:2 in assay diluent and added to the plate with known concentrations of TNFα and IL6 standards. Plates were incubated overnight at 4 °C, before washing five times with wash buffer. The TNFα or IL6 detection antibody (supplied with the kit) was added for 1 h at RT before washing five times and adding Avidin-HRP (supplied with the kit). Wells were incubated for 30 min and washed seven times. Substrate solution (TMB) (supplied with the kit) was added for 30 min at RT and the reaction was stopped by the addition of 2N H_2_SO4 (supplied with the kit). Wells were read on a 96-well plate reader (Spectramax Plus, Molecular Devices) at 450 nm and also then at 570 nm to subtract background absorbance.

### Allogeneic MLR

BMDC (K^b^ haplotype) were cultured from WT and TLR2^−/−^ bone marrow for 7 d as described previously, then activated by incubating with 100 μg/ml GL26 cell extract for 24 h (at 37 °C). CD4 and CD8 T cells were isolated using a magnetic activated cell sorter (MACS) from total splenocytes of Balb(c) mice (K^d^ haplotype) using mouse CD4 (L3T4) and CD8a (Ly-2) magnetic beads (Miltenyi Biotec). Total splenocytes were washed once with 15 ml MACS staining buffer (0.5% BSA in PBS with 2 mM EDTA, degassed), centrifuged for 15 min at 800*g*, and labeled with CD4 and CD8a magnetic beads (1:10) in MACS staining buffer (10^7^ cells/100 μl) for 15 min at 4 °C. Cells were then washed once more in MACS staining buffer and CD4^+^ and CD8a^+^ cells were positively selected using MACS LS columns in a Vario MACS sorter according to manufacturer's recommended protocol. 25,000 total responders (T cells, 1:1 ratio CD4:CD8a) were incubated in v-bottom 96-well plates (Corning) with increasing numbers (none, 1:100, 1:30, and 1:10) of allogeneic BMDCs (stimulators) in RPMI 1640 media supplemented with 10% FCS, 1% Pen-Strep for 3 d (at 37 °C). 1 × BrdU (Exalpha Biologicals Inc.) was added to the media for the final 24 h and BrdU incorporation into nascent DNA strands was assessed by ELISA (BrdU Cell Proliferation Assay). Relative proliferation of T cells compared with T cell proliferation in the absence of any stimulators was plotted for increasing ratios of stimulators (BMDC) compared with responders (T cells).

### Syngeneic MLR

WT and TLR2^−/−^ BMDC were differentiated as described above and loaded with 0.1 mg/ml GL26 cell extracts for 4 h. CD4^+^ and CD8a^+^ T cells were purified from total splenocytes from tumor-bearing mice 7 d after treatment with Flt3L and TK as described above. T cells were stimulated for 14 d with BMDC (1:10 ratio BMDC-GL26:T cell) loaded with GL26 tumor cell extract in the presence of 10 U/ml IL-2 to select for and expand tumor antigen specific T cell clones. Fresh BMDC-GL26 (1:10 ratio to T cells) were added after 7 d and fresh IL-2 (10 U/ml) was added every 4 d. On day 14, 1 × 10^5^ T cells (responders) were incubated per well in v-bottom 96-well plates (Corning) with increasing numbers (none, 1:100, and 1:30) of syngeneic BMDCs loaded with 0.1 mg/ml GL26 tumor antigen 24 h earlier (stimulators) in 200 μl RPMI 1640 media (CellGro) supplemented with 10% FCS, 1% Pen-Strep for 3 d. 1 × BrdU was added to the media for the final 24 h and BrdU incorporation into nascent DNA strands was assessed by ELISA (Exalpha Biologics Inc.) following the manufacturers protocol exactly.

### In Vitro TLR Activation Assay

An NFκB reporter plasmid driving firefly luciferase expression was used to determine signal transduction from TLR expressing HEK 293 cells as follows; HEK 293 cells were plated in 24-well plates at 50,000 cells per well and were transfected the next day with 50 ng pGL3-NFκB (Firefly Luciferase expressing reporter plasmid under the control of a NFκB response element), 5 ng pRL-TK (Renilla Luciferase expressing normalizing plasmid), and either 60 ng pCMV-TLR2 or 30 ng each of pCMV-TLR4 and pEF1α-MD2 using 1:2 ratio of DNA to JetPEI (Polyplus). All plasmids were purified with Endo-Free Maxiprep kits (Qiagen) and confirmed free of LPS using an LPS assay (Lonza). Media was removed 24 h later and replaced with fresh complete MEM. Cells were then stimulated for 6 h with 0.1 mg/ml GL26 cell lysate, GL26 cells treated with Ad-TK (+GCV), 100 ng/ml PAM3CSK4 (Invivogen), 1 μg/ml LPS (Invivogen), or an equal volume of endotoxin free medium as a negative control. TLR2-expressing 293 cells were incubated with the above stimuli in the presence or absence of glycyrrhizin (at a final concentration of 100 μM). Firefly activity was determined from cell lysates using a Dual Luciferase Reporter Assay kit (Promega) exactly as outlined by the manufacturers recommended protocol. Renilla Luciferase activity was also determined to normalize the activity of NFκB in each sample. Data are expressed as relative light units (RLU) by calculating the value of (FFLuc-Background)/(rLuc-Background).

### Generating HMGB1-Depleting Antibodies

Antibodies were custom generated by New England Peptides and raised in New Zealand White rabbits against the N-terminal 11 amino acids of HMGB1 (MGKGDPKKPRG) (see Figure S3) [39]. The peptide was conjugated to KLH using a C-terminal cysteine residue. Rabbits were immunized with the KLH-conjugated peptide in complete Freunds adjuvant and then boosted twice with the KLH-coupled peptide in incomplete Freunds adjuvant 14 d and 28 d after initial immunization. Serum was taken on day 35 and analyzed by ELISA (with a minimum of a 1:15,000 ELISA titer) and western blot (of GL26 cell extracts as described below). Serum was aliquoted and stored at −80 °C. IgG immunoglobulins were affinity purified using Protein G spin columns (Montage PROSEP-G, GE Healthcare) as described in the manufacturers technical datasheet. Briefly, the PROSEP-G column was pre-equilibrated with 10 ml binding buffer A by centrifuging at 500 × *g* for 5 min before loading 5 ml antibody (diluted 1:1 binding buffer A) and centrifuging at 150*g* for 20 min. The column was washed with 20 ml binding buffer A for 5 min to remove unbound contaminants and the antibody was eluted with 10 ml elution buffer B2 into 1.3 ml neutralizing buffer C. The affinity purified anti-HMGB1 antibody was used the same day in depletion studies (neat, 1 mg in 600 μl per mouse).

### SDS-PAGE and Western Blot Analysis of HMGB1 and β-Actin

1 ml media was removed from GL26 cells treated with Ad-TK and GCV 24, 48, and 72 h earlier (or mock infected controls) and centrifuged at 12,000 × *g* for 20 min to remove cell debris. We diluted the supernatants with SDS-PAGE loading buffer (100 mM Tris [pH 6.8], 20% v/v glycerol, 4% w/v SDS, 0.02% w/v bromophenol blue) with 50 mM DTT and boiled at 95 °C for 5 min. Denatured samples were loaded onto a 12% SDS-polyAcrylamide gel with 5% stacking gel. Electrophoresis of the samples was conducted at 130 V for 1.5 h and proteins were then transferred at 90 V for 1 h by wet transfer to a nitrocellulose membrane (GE Healthcare). The membrane was blocked with 5% milk in TBS + 0.05% Tween20 (Blotto) at RT for 1 h, then stained with either mouse anti-HMGB1 (Sigma H9537 1:1,000), rabbit anti-HMGB1 (custom, 1:1,000), or with mouse anti-β-Actin (Sigma A1978 1:1,000) for 1 h at RT. Membranes were washed three times in TBS with 0.05% Tween20 and HRP-conjugated rabbit anti-mouse immunoglobulins (DAKO P0260) were incubated in 5% blotto to detect HMGB1. Immunoreactivity was visualized by exposing Western blots to KODAK X-OMAT LS film and developed on a KODAK INDUSTREX M35 Processor. The density of immunostaining in each lane was determined using Adobe Photoshop 6.0.

### Inhibition of HMGB1 In Vivo with Glycyrrhizin and Depleting Anti-HMGB1 Antibodies

WT mice were challenged with 20,000 GL26 cells delivered into the striatum as described above and treated with Ad-Flt3L and Ad-TK (+GCV) or with an equal volume of saline (control) 17 d later. Glycyrrhizin (Calbiochem), was diluted fresh to a concentration of 100 mg/ml in 50 mM NaOH at 37 °C and pH was adjusted to pH 7.4 using 1M Tris-HCl. The solution was then filtered through a 0.22 μm syringe pump filter and 100 μl (10 mg glycyrrhizin) were administered to tumor-bearing mice by IP injection [40]. We administered glycyrrhizin 2, 5, and 10 d after injection of Ad-Flt3L and Ad-TK or saline. HMGB1-depleting immunoglobulins (custom antibody generated by New England Peptides) or rabbit IgG isotype control immunoglobulins (Sigma) were affinity purified (Montage, Millipore), tested for LPS contamination (Lonza), and injected in mice on day 19, 22, and 27 after tumor cell implantation (600 μg/mouse IP). Mice were monitored daily and euthanized when moribund. A Mantel log-rank test was used to determine significant differences in Kaplan-Meier survival curves.

### Treatment of Tumor Cell Lines with Ad-TK (+GCV), Radiation, or Chemotherapy

GL26, LLc1, GL261, or B16-F10 cells were seeded at a density of 2.5 × 10^5^ cells per flask. Cells were then treated with Ad-TK (+GCV), subjected to radiation, or treated with chemotherapy (temozolomide). Cells were infected with Ad-TK and treated with GCV as described above. As controls cells were infected with Ad0 at an MOI = 500. Cells were also mock infected as a control. 48 h later, supernatant was collected to assess HMGB1 release by Western blot or HMGB1 specific ELISA. To assess the effects of radiation on HMGB1 release from tumor cell lines, cells were irradiated with 20 Gy of gamma radiation for 30 min at room temperature. As controls, cells were left at room temperature for 30 min. 72 h later, supernatant was collected to assess HMGB1 release by Western blot or HMGB1 specific ELISA. To assess whether tumor cell lines release HMGB1 following treatment with chemotherapy, cells were incubated with temozolomide at a final concentration of 200 μM [41]. 48 h later, supernatant was collected to assess HMGB1 release by Western blot or HMGB1 specific ELISA.

### Statistical Analysis

Due to difficulties in verifying normality of distribution when the sample size is small, we used the following nonparametric tests, i.e., Mann-Whitney *U*-test was used to determine whether two independent samples (cohorts) came from the same population, and the Kruskal-Wallis H test followed by Dunn's test was used to determine if several independent samples came from the same population; these tests were calculated using NCSS 2007 software (NCSS). Curve inequality (phagocytosis and MLR experiments) was assessed using the randomization test; also implemented on NCSS 2007 software (NCSS). All data are represented using scatter plots. The effect size reported for the Mann-Whitney *U*-test and the Kruskal-Wallis H test is partial η^2^ (eta-squared) and was calculated using MINSIZE2 (kindly provided by David T. Morse, Department of Counseling & Education Psychology, Mississippi State University, Mississippi State, Mississippi, USA [42–45]). Kaplan-Meier survival curves were analyzed using the Mantel log-rank test and the effect size is expressed as the median survival ratio (MSR); this was calculated using GraphPad Prism version 3.00 (GraphPad Software). *p*-Values of less than 0.05 were considered significant. The statistical tests used are indicated within the figure legends. Sample sizes were calculated to detect differences between groups with a power of 80% at a 0.05 significance level using PASS 2008 (Power and sample size software, NCSS).

## Results

### Intratumoral Expression of Flt3L and TK Eliminates Brain Tumors in a T Cell-Dependent Manner

**Figure 1:**
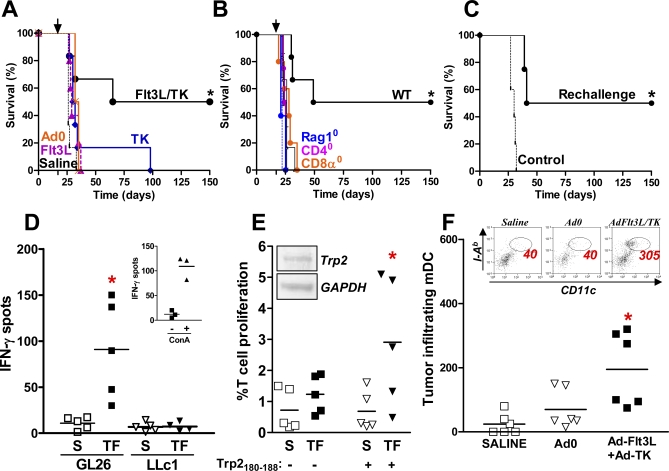
Flt3L and TK Recruits DC into Brain Tumors and Mediates T Cell–Dependent Brain Tumor Regression (A) GL26 cells were implanted in the striatum of C57BL/6 mice. Tumor-bearing mice were treated 17 d later with Ad-Flt3L and Ad-TK (black solid line, *n* = 6), Ad-TK (blue solid line, *n* = 6), Ad-Flt3L (purple solid line, *n* = 5), Ad0 (orange solid line, *n* = 4), or saline (black broken line, *n* = 6). Treatment with Ad-Flt3L and Ad-TK significantly improved survival; *, *p* < 0.05 versus saline (Mantel log-rank test). (B) GL26 cells were implanted in the striatum of WT (black, *n* = 6), or *Rag1* ^−/−^ (Rag1^0^, blue , *n* = 5), *CD4* ^−/−^ (CD4^0^, purple, *n* = 4), or *CD8* ^−/−^ (CD8^0^, orange, *n* = 5) knockout mice. 17 d later, mice were treated with Ad-Flt3L and Ad-TK (solid lines) or saline (broken lines). Treatment with Ad-Flt3L and Ad-TK significantly improved survival of WT animals when compared to all other strains; (*, *p* < 0.05; Mantel log-rank test). (C) Long-term survivors (>150 d post-tumor cell implantation) were rechallenged with GL26 cells in the contralateral hemisphere (solid line, *n* = 4). Naïve, age matched C57BL/6 mice were also implanted with GL26 cells as a control (broken line, *n* = 5). Only long-term survivors showed increased survival following tumor rechallenge (*, *p* < 0.05 versus saline; Mantel log-rank test). (D) Tumor antigen specific, IFNγ-producing T lymphocytes were quantified using an IFNγ ELISPOT. GL26 cells were implanted in the brains of C57BL/6 mice and treated 17 d later with Ad-Flt3L and Ad-TK (TF) or Saline (S). T cells were isolated and incubated with DC loaded with either GL26 tumor antigen (GL26) or LLc1 tumor antigen (LLc1) before quantification of IFNγ production. Inset: Concanavalin A (ConA)-stimulated splenocytes (+) are shown as a positive control. *, p < 0.05 versus saline (Mann-Whitney *U*-test). (E) A T cell proliferation assay was used to determine the percentage of T cells that recognize the tumor antigen Trp2_180–188_. GL26 cells were implanted in the brains of mice, and treated after 17 d with Ad-Flt3L and Ad-TK or with saline. Splenocytes were isolated 7 d later and stained with CFSE, then incubated with (+) or without (−) Trp2_180–188_. CD8+ T cell proliferation was calculated using flow cytometry by quantifying the percentage of cells that had divided (sequential dilution of CFSE fluorescence). *, p < 0.05 versus saline (Mann-Whitney *U*-test). Inset: Reverse transcriptase PCR confirms the expression of Trp2 mRNA by GL26 cells. GAPDH primers were used as a control. (F) GL26 cells were implanted in the brain of C57BL/6 mice and treated 17 d later with Ad-Flt3L and Ad-TK, Ad0, or saline. The total number of tumor-infiltrating DCs (CD11c^+^ CD45^High^ MHC II^+^) was quantified by flow cytometry. *, p < 0.05 versus saline (Kruskal-Wallis test followed by Dunn's test). Representative dot plots display tumor-infiltrating CD45^+^ immune cells stained with CD11c (*x*-axis) and MHC II (I-A^b^; *y*-axis). DCs were identified as (CD11c^+^ CD45^High^ MHC II^+^). The total number of tumor-infiltrating mDC is indicated for each plot.

To uncover the role of TLR signaling in brain tumor regression and immunological memory, we developed a syngeneic, intracranial mouse glioma model; treatment consisted of intratumoral delivery of Ads expressing Flt3L and TK (Ad-TK + Ad-Flt3L), followed by systemic delivery of GCV. We observed long-term survival after treatment (>150 d) in 50% of tumor-bearing WT mice treated with Ad-TK + Ad-Flt3L (*, *p* < 0.05 versus saline; MSR 4.1; Figure 1A) and failure to improve survival in *Rag1^−/−^*, *CD4^−/−^*, or *CD8a^−/−^* mice (all on C57BL/6 background) compared with WT controls (Figure 1B), indicating T cell-dependent tumor regression. This treatment also elicited anti-GBM immunological memory (*, *p* < 0.05 versus saline; MSR 3.2; Figure 1C). To investigate whether clonal expansion of tumor antigen specific T lymphocytes occurred in response to Flt3L and TK treatment, we pulsed BMDCs with GL26 brain tumor cell extracts or LLc1 lung carcinoma cell extracts, and incubated these BMDCs with T lymphocytes isolated from tumor-bearing mice. The frequency of T cell precursors that released IFNγ in response to specific tumor antigens was increased in mice implanted with GBM and treated with Flt3L and TK (TF) (*, *p* < 0.05; Figure 1D). Analysis of the effect size revealed a partial eta-squared (η^2^) value of 0.69, considered to be a large effect [44–46]. Trp2_180–188_, a known H2-K^b^-restricted melanoma tumor antigen shared by glioma tumors such as GL26 cells [47,48], also induced proliferation of T lymphocytes from glioma-bearing mice treated with Flt3L and TK (*, *p* < 0.05 versus saline; η^2^ = 0.43; Figure 1E) confirming that clonal expansion of T lymphocytes against a specific glioma antigen occurs in response to treatment.

In order to mount a specific antitumor immune response, DCs need to infiltrate the tumor mass where they can phagocytose tumor antigen, and migrate to the cervical dLN to elicit clonal expansion of antitumor specific T cells. The number of mDCs (CD11c^+^; CD45^+^; MHC II^+^) isolated from the tumors increased almost 10-fold in WT C57BL/6 mice 7 d after treatment with Flt3L and TK compared with saline treated controls (*, *p* < 0.05 versus saline; η^2^ = 0.54; Figure 1F).

### Tumor-Infiltrating DCs, in Response to Flt3L and TK Treatment, Originate from the Bone Marrow

**Figure 2:**
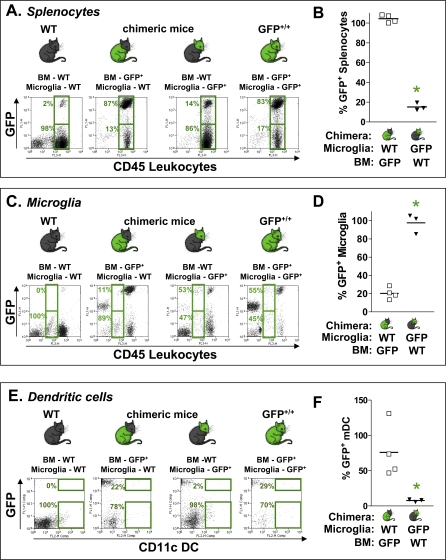
Tumor-Infiltrating DC Are Bone Marrow Derived (A–F) Bone marrow chimeric mice were used to assess the origin of CD11c^+^ mDC infiltrating into the tumor. (A,C,E) WT mice are depicted on the left panels; chimeras are as follows: WT mice adoptively transferred with GFP^+/+^ bone marrow; or GFP^+/+^ mice adoptively transferred with WT bone marrow; GFP^+/+^ mice are depicted on the right panels. GL26 cells were implanted in all mice 10 wk after irradiation and treated 17 d later with Ad-Flt3L and Ad-TK. 7 d after the treatment, tumor-infiltrating immune cells and splenocytes were isolated; flow cytometry was performed to detect total GFP^+^ splenocytes (A and B), GFP^+^ tumor-infiltrating microglia (C and D), or GFP^+^ tumor infiltrating mDC (E and F). Representative dot plots show (A) GFP^+^ (upper boxes, GFP^+^, CD45^hi^) or GFP^−^ splenocytes (lower boxes, GFP^−^, CD45^hi^); (C) GFP^+^ microglia (upper boxes, GFP^+^, CD45^Int^) or GFP^−^ microglia (lower boxes, GFP^−^, CD45^Int^); and (E) GFP^+^ mDC (upper boxes, GFP^+^, CD11c^+^) or GFP^−^ mDC (lower boxes, GFP^−^, CD11c^+^) infiltrating into the brain tumors. The normalized percentage of GFP^+^ splenocytes (B), tumor-infiltrating GFP^+^ microglia (D), and tumor-infiltrating GFP^+^ mDC (F) are shown for each type of chimeric mouse (*, p < 0.05; Mann-Whitney *U*-test).

To investigate whether CD11c^+^ tumor-infiltrating immune cells originate from bone marrow, we generated bone marrow chimeric mice. To do so, we adoptively transferred GFP^+^ bone marrow into irradiated WT mice, or conversely transplanted WT bone marrow into irradiated GFP^+^ mice. 10 wk after adoptive transfer, mice were implanted with GL26 tumor cells in the striatum. We treated tumor-bearing chimeric mice with Ad-Flt3L and Ad-TK 17 d post-tumor implantation and characterized the origin of tumor-infiltrating CD11c^+^ mDC 7 d after treatment. To visualize total splenocytes or tumor-infiltrating microglia, live immune cells were gated (FSC versus SSC), and CD45 was plotted against GFP fluorescence (Figure 2A and 2C). To visualize tumor-infiltrating mDC, live CD45^+^ immune cells were gated and CD11c was plotted against GFP (Figure 2E). The majority of splenocytes in the chimeric mice exhibited the phenotype of the donor mice (*, *p* < 0.05; η^2^ = 0.75; Figure 2A and 2B). Microglial cells, or any brain resident immune cells are radio-resistant because of their low mitotic rate [49], and thus most microglia exhibited the phenotype of recipient mice (*, *p* < 0.05; η^2^ = 0.75; Figure 2C and 2D). If CD11c^+^ immune cells that infiltrate into the brain tumor originate from the bone marrow, they would exhibit the phenotype of the adoptively transferred bone marrow; if they were to originate from CNS sources they would retain the phenotype of the recipient mice. In chimeric mice generated by adoptively transferring bone marrow from GFP^+/+^ mice into irradiated WT mice, 22.23% (±5.64%) of tumor-infiltrating mDC were GFP^+^. In chimeric mice generated by adoptively transferring bone marrow from WT mice into irradiated GFP^+/+^ mice; 2.24% (±0.32%) of tumor-infiltrating mDC were GFP^+^ (Figure 2E). Since 0% of mDC from WT mice displayed the phenotype of GFP^+^ CD11c^+^ mDC, and 29.27% of cells from GFP^+/+^ mice displayed the phenotype of GFP^+^ CD11c^+^ mDC (Figure 2E), we calculated the normalized percentage of GFP^+^ mDC infiltrating into the tumors of each chimeric mouse from the data shown in Figure 2E using the following equation: %normalized GFP^+^ mDC = [%GFP^+^ mDC in the chimera − 0% (background)]/[29.27%(GFP^+^ mDC in GFP^+/+^ mice) − 0% (background)] × 100. We found that 75.95% (±19.25%) of tumor-infiltrating mDC in chimeric mice generated by adoptively transferring bone marrow from GFP^+/+^ mice into irradiated WT mice (white) could be conclusively identified as originating from bone marrow. This was significantly greater (*, *p* < 0.05; η^2^ = 0.75) than the 7.65% ± 1.08% GFP^+^ mDC encountered in the chimeric mice generated by adoptively transferring bone marrow from WT mice into irradiated GFP^+/+^ mice (black) (Figure 2F). Therefore, our data suggest that the vast majority of CD11c^+^-infiltrating immune cells in Ad-Flt3L and Ad-TK-treated brain tumors are bone marrow-derived mDC.

### TLR2 Expression on BMDC Is Necessary for Tumor Regression

**Figure 3:**
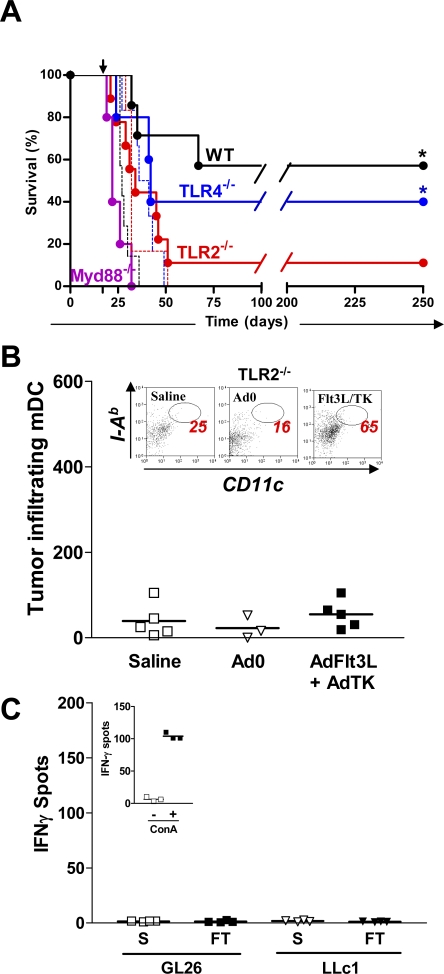
TLR2 Signaling Mediates T Cell-Dependent Brain Tumor Regression (A) TLR2 and Myd88 are required for Ad-Flt3L and Ad-TK mediated brain tumor regression. GL26 cells were implanted into C57BL/6, Myd88^−/−^, *TLR4^−/−^*, and *TLR2^−/−^* mice. 17 d later C57BL/6 (solid black, *n* = 7), Myd88^−/−^ (solid purple, *n* = 5), *TLR4^−/−^* (solid blue, *n* = 5), and *TLR2^−/−^* mice (solid red, *n* = 9) were treated with an intratumoral injection of Ad-Flt3L and Ad-TK (solid lines). As controls, C57BL/6 (dashed black, *n* = 7), *TLR4^−/−^* (dashed blue, *n* = 4), and *TLR2^−/−^* mice (dashed red, *n* = 6) were treated with saline. *, *p* < 0.05 versus saline; Mantel log-rank test. (B) GL26 cells were implanted in the striatum of *TLR2* ^−/−^ mice and treated 17 d later with Ad-Flt3L and Ad-TK, Ad0, or saline. Flow cytometry was used to quantify the total number of tumor-infiltrating DCs (CD11c^+^ CD45^High^ MHC II^+^). Kruskal-Wallis test. Inset: Representative dot plots display tumor-infiltrating CD45+ immune cells stained for CD11c (*x*-axis) and MHC II (I-A^b^; *y*-axis). DCs were identified as (CD11c^+^ CD45^High^ MHC II^+^). The total number of tumor-infiltrating mDC are indicated for each plot. (C) Treatment of tumor-bearing TLR2^−/−^ mice with Ad-Flt3L and Ad-TK fails to induce clonal expansion of tumor antigen specific T lymphocytes. GL26 cells were implanted in the brains of TLR2^−/−^ mice and treated with Ad-Flt3L and Ad-TK (FT) or saline (S) after 17 d. T cells were purified from the spleens 7 d later and incubated with WT DC loaded with GL26 tumor extracts (GL26) or with syngeneic LLc1 carcinoma cell extracts (LLc1) before quantification of IFNγ producing cells by ELISPOT. Mann-Whitney *U*-test. Inset: Concanavalin A (ConA) stimulated splenocytes (+) are also shown as a positive control.

To determine if TLRs were involved in mediating the efficacy of the combined antiglioma treatment, we investigated whether Myd88 knockout mice would respond to our therapy, as Myd88 is required for downstream signaling from every TLR except TLR3 [12,50]. Treatment with Flt3L and TK failed to eliminate brain tumors in *Myd88* ^−/−^ mice (Figure 3A) suggesting that one or more TLR signaling pathways are required for T cell-dependent tumor regression in this model. Importantly, TLR2 and TLR4 have recently been demonstrated to play a role in models of brain inflammation [51–53], and several endogenous TLR2 and TLR4 ligands such as gp96, HMGB1, HSP60, HSP70, and hyaluronan have been identified in various tumors [54–58]. Thus, we tested the role of TLR2 and TLR4 in TK- and Flt3L-mediated glioma regression. Analysis of brain sections by immunohistochemistry with antibodies specific for Flt3L and TK confirmed that both therapeutic transgenes were efficiently expressed in the brains of TLR2^−/−^ and TLR4^−/−^ mice (unpublished data). Treatment with Flt3L and TK failed in TLR2^−/−^ mice, whereas it significantly improved survival of TLR4^−/−^ mice (*, *p* < 0.05 versus saline; MSR 1.55; Figure 3A). These data demonstrate that TLR2, but not TLR4, mediates T cell-dependent brain tumor regression in this model. Unlike WT mice (Figure 1F) tumor-infiltrating mDCs did not increase in Flt3L- and TK-treated *TLR2* ^−/−^ mice (Figure 3B); indicating that the migration of DCs into the tumor mass in response to TK and Flt3L treatment is TLR2 dependent. Further, the frequency of tumor antigen specific T lymphocytes did not increase in treated *TLR2* ^−/−^ mice (Figure 3C). Treatment with Flt3L and TK induced proliferation of CD4^+^ (*, *p* < 0.05 versus saline; η^2^ = 0.76; Figure S4A) and CD8α^+^ T lymphocytes (*, *p* < 0.05 versus saline; η^2^ = 0.74; Figure S4B) in the draining LN of tumor-bearing WT mice, but not their TLR2^−/−^ counterparts.

**Figure 4:**
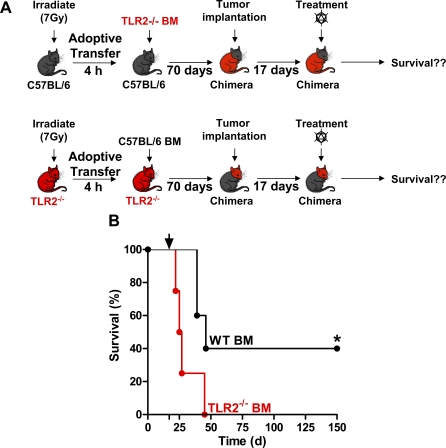
TLR2 Signaling on Bone Marrow-Derived Immune Cells and Not Microglia Is Necessary for Brain Tumor Regression (A) Schematic describing the generation of chimeric mice and subsequent implantation of tumors and treatment. WT irradiated C57BL/6 mice were adoptively transferred with *TLR2^−/−^* bone marrow (top); *TLR2^−/−^* irradiated mice were adoptively transferred with WT bone marrow (bottom). 70 d later chimeric animals were implanted with tumor cells and treated with Ad-Flt3L and Ad-TK, 17 d post-tumor implantation. (B) Kaplan-Meier curve depicting survival when chimeric mice were challenged with intracranial GBM and treated with Ad-Flt3L and Ad-TK (wt type bone marrow, black line, *n* = 5; TLR2−/− bone marrow, red line, *n* = 4). (*, *p* < 0.05, Mantel log-rank test).

**Figure 5:**
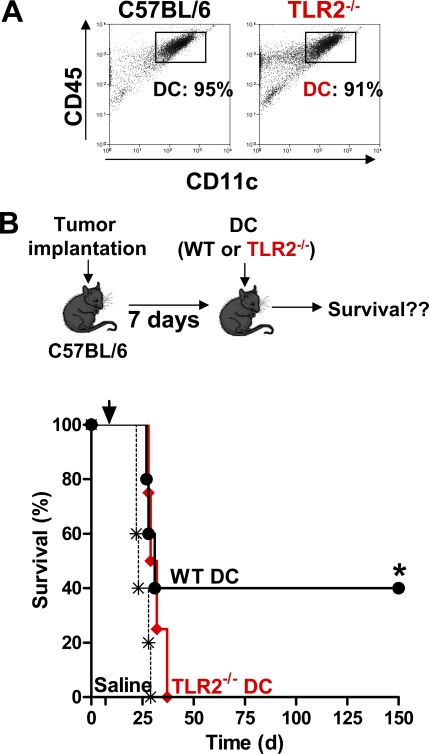
TLR2 Signaling on BMDCs Is Necessary for Brain Tumor Regression (A) CD11c^+^ mDC were generated from bone marrow of C57BL/6 mice (left) or TLR2^−/−^ mice (right) and identified using flow cytometry. (B) GL26 cells were implanted in the brains of WT mice, and 7 d later mice were treated with an intratumoral injection of WT BMDCs loaded with GL26 tumor extracts (black, *n* = 5), TLR2^−/−^ BMDCs loaded with GL26 tumor extracts (red, *n* = 4), or saline (dashed black, *n* = 5). *, *p* < 0.05 versus saline; Mantel log-rank test.

TLR2 signaling in DCs mediates upregulation of costimulatory molecules and facilitates clonal expansion of antigen specific T lymphocytes [59,60]. Thus, we wished to investigate whether TLR2 signaling on tumor-infiltrating DCs mediated T cell-dependent tumor regression in our model. To do this, the following experiments were performed in bone marrow chimeric mice: (1) irradiated *TLR2* ^−/−^ mice were adoptively transferred with WT bone marrow, and (2) WT mice were irradiated and were adoptively transferred with TLR2^−/−^ bone marrow (Figure 4A); both sets of chimeric animals were implanted with tumors and treated with Ad-Flt3L and Ad-TK. Tumor regression was only observed in irradiated TLR2^−/−^ mice adoptively transferred with WT bone marrow (*, *p* < 0.05; MSR 1.8; Figure 4B); this strongly suggests that TLR2 expression is necessary on bone marrow-derived, tumor-infiltrating immune cells. Next, we wished to determine whether TLR2 signaling exclusively on BMDCs was sufficient to induce brain tumor regression. To this end, we implanted BMDC (Figure 5A) loaded with GL26 extracts into the tumor mass. Intratumoral delivery of WT DC pulsed with GL26 tumor extract mediated regression of intracranial brain tumors in WT mice (*, *p* < 0.05 versus saline; MSR 1.2; Figure 5B), whereas *TLR2* ^−/−^-derived DC pulsed with GL26 tumor extract failed to induce brain tumor regression (Figure 5B). Together, our results demonstrate that endogenous activation of TLR2 on tumor-infiltrating BMDCs is necessary for initiating T lymphocyte clonal expansion and tumor regression.

### TLR2 Is Necessary for DC Activation and Antigen Presentation

**Figure 6:**
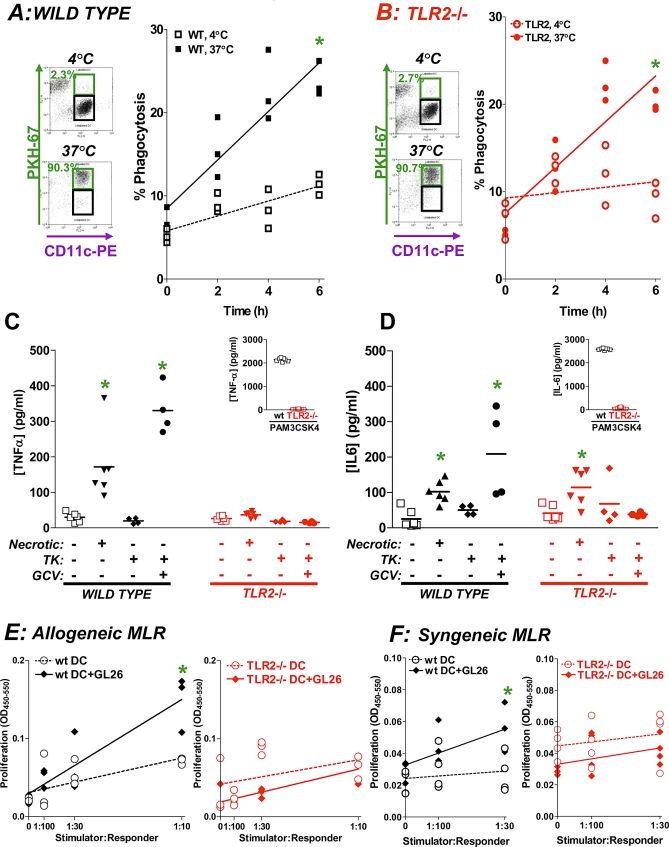
TLR2 Is Necessary for DC Activation and Antigen Presentation but Not for Tumor Phagocytosis In Vitro Extracts were made of GL26 cells labeled with PKH-67 and incubated with WT (A) or TLR2^−/−^ (B) BMDC for 16 h either at 4 °C or 37 °C. The percentage of CD11c+ DC that phagocytosed labeled GL26 cell extracts (green boxes) was quantified by flow cytometry. The scatter plots depict the rate of phagocytosis of PKH67 labeled GL26 cells by WT (A) and TLR2^−/−^ (B) DC every 2 h for 6 h at 4 °C and 37 °C. *, p < 0.05 versus 4 °C (randomization test). Secretion of TNFα (C) and IL6 (D) from WT DC (black) or TLR2^−/−^ DC (red) was determined by ELISA after incubation with: GL26 cell extracts, media from GL26 cells infected with Ad-TK (with and without GCV), or media (unstimulated controls). Inset: Pam3CSK4-treated DCs (positive control). *, p < 0.05 versus unstimulated controls (Kruskal-Wallis test followed by Dunn's test). (E) An allogeneic mixed leukocyte reaction (MLR) was used to determine whether DC could stimulate T cell proliferation. DC (stimulators) from C57BL/6 mice (left panel, black) or TLR2^−/−^ mice (right panel, red) were incubated with GL26 cell extracts before adding to allogeneic naïve Balb(c) T cells (responders). BrdU incorporation into DNA was measured to determine relative proliferation of T cells. BMDC that were not loaded with GL26 cell extracts were used as a control for background T cell proliferation (dashed lines). *, p < 0.05 versus unloaded DCs; randomization test. (F) A syngeneic MLR was used to determine if TLR2 activation on DCs can induce tumor antigen specific T cell proliferation. DC (stimulators) from WT mice (left panel, black) or TLR2^−/−^ mice (right panel, red) were incubated with GL26 cell extracts and added to tumor antigen specific T cells (responders). BrdU incorporation into DNA was measured to determine relative proliferation of T cells. BMDC that were not loaded with GL26 cell extracts were used as a control for background T cell proliferation (dashed lines). *, p < 0.05 versus unloaded DCs; randomization test.

Previous studies have shown that necrotic cell extracts can stimulate DC activation [61]. We hypothesized that tumor cell necrosis could release endogenous TLR2 ligands and thus activate DC. Our results show that necrotic GL26 and other tumor cells induced TLR2 dependent; TLR4 independent, activation of NFκB-signaling (Figure S5), suggesting that endogenous TLR2 ligand(s) released upon cell death could activate the TLR2-dependent NFκB signal transduction pathway. Activation of TLR signaling pathways on DC can differentially modulate phagocytosis of tumor cell fragments, costimulatory molecule expression, and production of inflammatory cytokines [62]. We investigated whether endogenous TLR2 activity on BMDC was important for phagocytosis of tumor cell remnants, proliferation of tumor antigen specific T lymphocytes, and expression of inflammatory cytokines. Both WT and *TLR2^−/−^* BMDC phagocytosed labeled GBM cell lysates (Figure 6A and 6B). DC activation in response to GL26 tumor extracts was dependent on TLR2 expression; we observed that the release of the pro-inflammatory cytokines TNFα and IL6 was elevated when WT BMDC were incubated with either media from GL26 cells killed with Ad-TK and GCV or necrotic GL26 cells (*, *p* < 0.05 versus mock; Figure 6C, η^2^ = 1.0; Figure 6D, η^2^ = 0.73). TNFα release was not increased from *TLR2* ^−/−^ DC incubated with either media from GL26 cells killed with Ad-TK (+GCV) or necrotic GL26 cells (Figure 6C). IL6 release did not increase in TLR2^−/−^ DC incubated with media from GL26 cells treated with Ad-TK (+GCV) (Figure 6D). We next investigated whether TLR2 signaling was required to induce T cell proliferation in both allogeneic and syngeneic, antigen-specific mixed lymphocyte reaction assays (MLR). WT BMDC (*, *p* < 0.05; Figure 6E) but not *TLR2^−/−^* BMDC (Figure 6E) promoted allogeneic T lymphocyte proliferation. Next we determined that only WT BMDC, but not *TLR2* ^−/−^ BMDC pulsed with GL26 cell extracts induced proliferation of tumor antigen specific T lymphocytes (*, *p* < 0.05, Figure 6F). Thus, TLR2 signaling on DC in response to GL26 tumor cells is necessary for DC activation and presentation of tumor antigen to T lymphocytes.

### HMGB1 Is the Endogenous Ligand That Mediates TLR2-Dependent Glioma Tumor Regression.

**Figure 7:**
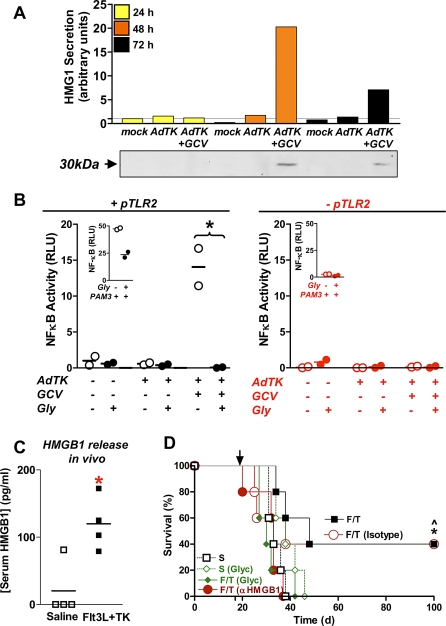
HMGB1 Is Secreted from Dying Tumor Cells and Is Required for Ad-Flt3L and Ad-TK Mediated Brain Tumor Regression (A) GL26 cells were infected with Ad-TK and incubated with 25 μM GCV. A western blot was performed using an antibody specific for HMGB1. The graph displays a quantification of the total amount of HMGB1 in the media of cells 24 h (yellow bars), 48 h (orange bars), or 72 h (black bars) after GCV treatment. (B) GL26 cells were incubated with Ad-TK (with and without GCV and glycyrrhizin), and media was overlaid on HEK293 reporter cells transfected with either a plasmid encoding TLR2 (+pTLR2; black) or with a control plasmid (-pTLR2; red). NFκB activity was determined by quantifying the activity of Firefly Luciferase (under the control of the NFκB promoter), *, p < 0.05 (Kruskal-Wallis followed by Dunn's test). Inset: cells were incubated with 100 ng/ml PAM3 (PAM3CSK4) as a positive control. (C) The levels of HMGB1 in mouse serum were quantified by ELISA 7 d after treatment of brain tumors with Ad-Flt3L and Ad-TK. *, p < 0.05 versus saline (Mann-Whitney *U*-test). (D) GL26 cells were implanted into C57BL/6 mice (*n* = 5 mice/treatment group) and 17 d later were treated with saline, or with AdFlt3L and Ad-TK (F/T). Glycyrrhizin (Glyc), HMGB1-depleting (αHMGB1), or rabbit IgG isotype control antibodies were administered IP 2 d, 5 d, and 10 d after treatment, *, *p* < 0.05 Ad-Flt3L and Ad-TK versus Ad-Flt3L and Ad-TK + glycyrrhizin; ^*p* < 0.05 Ad-Flt3L and Ad-TK + isotype versus Ad-Flt3L and Ad-TK + αHMGB1; Mantel log-rank test.

We hypothesized that endogenous TLR2 ligands would also be released from GL26 cells treated with Ad-TK + GCV. In turn, endogenous TLR2 ligands could play a role in tumor regression. Interestingly, it was recently shown that HMGB1 released from lymphoma, breast, and colon tumor cells enhances tumor regression induced by standard chemotherapeutic agents by activating TLR4 [9]. HMGB1 can also activate TLR2 in addition to TLR4 and differential TLR activation appears to depend on the particular tumor model used [58]. Therefore, we hypothesized that HMGB1 could be an endogenous TLR2 ligand released from TK + GCV treated GL26 cells. Our results show an increase in extracellular HMGB1 (approximately 30 kDa MW detected by western blot) released from Ad-TK + GCV treated GL26 tumor cells in vitro 48 h and 72 h after treatment (Figure 7A). This event occurred concomitantly with the binding of Annexin V to phosphatidylserine and the formation of a typical DNA ladder, which is the hallmark of apoptotic cells (Figure S6). To investigate whether HMGB1, released from dying GL26 tumor cells (Ad-TK + GCV treated), was responsible for TLR2 activation in vitro, we inhibited HMGB1 binding using glycyrrhizin, a known antagonist of HMGB1 [40,63]. Glycyrrhizin completely blocked TLR2-dependent NFκB activation by supernatants from dying (Ad-TK + GCV treated) GL26 cells (*p < 0.05; η^2^ = 0.75; Figure 7B) indicating that HMGB1 secreted from GL26 cells stimulates TLR2 signaling. We next investigated whether HMGB1 was released into the serum of GBM-bearing mice treated with Ad-TK + GCV and Flt3L in vivo. HMGB1 was increased in the serum of tumor-bearing mice 7 d after treatment with Flt3L and TK (*p < 0.05; η^2^ = 0.49; Figure 7C), suggesting that HMGB1 released from dying tumor cells might be responsible for activating TLR2 on DC in vivo and subsequent T cell-dependent tumor regression.

To test the hypothesis that HMGB1 secreted from GBM-bearing mice after treatment with Flt3L plus TK (GCV) mediates TLR2 signaling and T cell-dependent brain tumor regression in vivo, we blocked circulating HMGB1 in vivo using glycyrrhizin or specific anti-HMGB1 immunoglobulins. HMGB1 antibodies were generated by us, using the N-terminal 11 amino acids of the HMGB1 peptide sequence previously described (Figure S3A) [39]. The anti-HMGB1 antibodies, recognized a single ∼30-kDa band from GL26 cell lysates by western blot (Figure S3B). Inhibition of extracellular HMGB1 activity in vivo using either blocking antibodies or glycyrrhizin resulted in the complete failure of our therapy (Figure 7D). Together, our data provide compelling evidence that HMGB1, released from GBM tumors treated with Ad-Flt3L and Ad-TK in vivo, elicits TLR2 signaling that results in recruitment of BMDC into the tumor mass, with concomitant induction of antitumor specific T cell clonal expansion, long-term survival, and immunological memory.

### HMGB1 Is Released from Several Tumor Cell Lines When Treated with Either Ad-TK (+GCV), Radiation, or Chemotherapy

**Figure 8:**
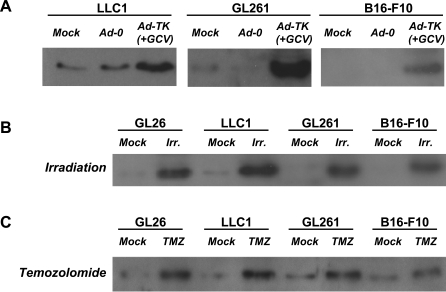
Western Blot Analysis Reveals HMGB1 Is Released from GL26, LLc1, GL261, and B16-F10 Cell Lines Following Treatment with Ad-TK (+GCV), Irradiation, or Temozolomide (TMZ) (A) LLc1, GL261, or B16-F10 cells were infected with Ad-TK or Ad-0 at an MOI = 500. Noninfected cells were used as control. Ad-TK treated cells were also incubated with 25 μM GCV. 48 h later, supernatant was collected and HMGB1 release was assessed by western blotting. (B) GL26, LLc1, GL261, or B16-F10 cells were irradiated with 20 Gy for 30 min. Nonirradiated cells were used as control. 72 h later, supernatant was collected and HMGB1 release was assessed by western blotting. (C) GL26, LLc1, GL261, or B16-F10 cells were incubated with temozolomide (TMZ) (200 μM). Control cells were incubated without TMZ (mock). 48 h later, supernatant was collected and HMGB1 release was assessed by western blotting.

**Figure 9:**
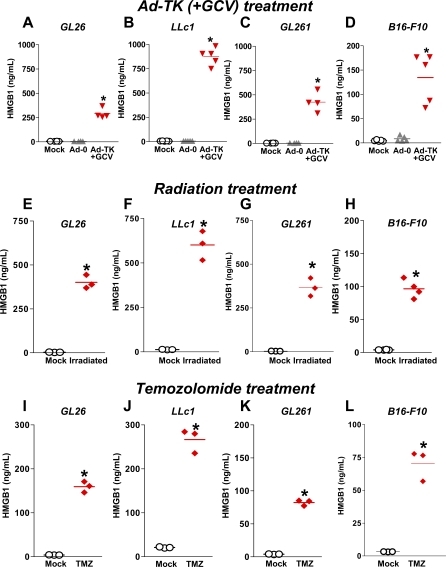
HMGB1 Is Released into the Supernatant of GL26, LLc1, GL261, and B16-F10 Tumor Cell Lines in Response to Treatment with Ad-TK (+GCV), Irradiation, or Temozolomide (A–D) GL26, LLc1, GL261, or B16-F10 cells were infected with Ad-TK or Ad-0. Noninfected cells were used as control (mock). Ad-TK treated cells were incubated with 25 μM GCV. 48 h later, supernatant was collected, and HMGB1 release was assessed by ELISA. Treatment with Ad-TK (+GCV) significantly increased HMGB1 release when compared to corresponding mock and Ad0 treatment groups from each cell line, *, *p* < 0.05 versus mock and Ad-0 (Kruskal-Wallis test followed by Dunn's test). (E–H) GL26, LLc1, GL261, or B16-F10 cells were irradiated with 20 Gy for 30 min. Nonirradiated cells were used as controls (mock). 72 h later, supernatant was collected, and HMGB1 release was assessed by ELISA. Treatment with irradiation significantly increased HMGB1 release when compared to corresponding control groups from each cell line, *, p < 0.05 versus mock (Mann-Whitney *U*-test). (I–L) GL26, LLc1, GL261, or B16-F10 cells (2.5 × 10^5^ cells per flask) were incubated with or without temozolomide (TMZ) (200 μM). 48 h later, supernatant was collected, and HMGB1 release was assessed by ELISA. Treatment with TMZ significantly increased HMGB1 release when compared to control groups from each cell type. *, p < 0.05 versus mock (Mann-Whitney *U*-test).

To assess whether HMGB1 release is specific to Ad-TK (+GCV)-induced GL26 cell-killing or a more general phenomenon, we treated GL26 (glioma), LLc1 (lung carcinoma), GL261 (glioma), or B16-F10 (melanoma) cells in vitro with three different cell-killing modalities currently in use in the clinic or clinical trials for brain tumors: either Ad-TK (+GCV), radiation, or chemotherapy (temozolomide). Western blot analysis of cell culture supernatants revealed increased levels of HMGB1 following treatment with either Ad-TK (+GCV), radiation, or temozolomide (Figure 8). We then performed a HMGB1-specific ELISA to quantitate the levels of HMGB1 release from GL26, LLc1, GL261, or B16-F10 cells in vitro following treatment with either Ad-TK (+GCV), radiation, or temozolomide. Figure 9A–9D reveals statistically significant increases (*p < 0.05 versus mock and Ad0) in HMGB1 levels in the supernatants of GL26 (η^2^ = 0.67), LLc1 (η^2^ = 0.68), GL261 (η^2^ = 0.73), or B16-F10 cells (η^2^ = 0.72) in response to Ad-TK (+GCV). Irradiation (Figure 9E–9H) and temozolomide (Figure 9I–9L) treatments also caused a significant increase (*, *p* < 0.05) in the levels of HMGB1 released from GL26 (η^2^ = 0.60 and 0.63, respectively), LLc1 (η^2^ = 0.60 for both treatments), GL261 (η^2^ = 0.60 for both treatments), and B16-F10 tumor cells (η^2^ = 0.67 and 0.60, respectively). Taken together, these data strongly suggest that HMGB1 release following treatment of tumor cells with a variety of cytotoxic agents is a wide-spread phenomenon regardless of tumor cell type or tumor cell killing approach.

### Treatment of Mice Bearing a Second Variant of Intracranial Glioma (GL261) or Melanoma (B16-F10) Tumors with Ad-Flt3L and Ad-TK (+GCV) Results in Long-Term Survival and Increased Serum Levels of HMGB1

**Figure 10:**
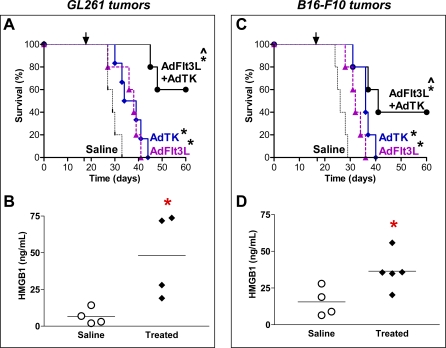
Treatment of Animals Bearing Intracranial Glioma (GL261) or Melanoma (B16-F10) with Ad-Flt3L and Ad-TK (+GCV) Results in Long-Term Survival and Increased Serum Levels of HMGB1 (A) GL261 cells were implanted into the striatum of C57/Bl6 mice. 17 d later animals were treated with Ad-Flt3L (*n* = 5), Ad-TK (+GCV) (*n* = 6), Ad-Flt3L plus Ad-TK (+GCV) (*n* = 5), or saline (*n* = 5). Treatment with Ad-Flt3L and Ad-TK significantly improved survival when compared to saline (*, *p* < 0.05; Mantel log-rank test), Ad-Flt3L, or Ad-TK alone treatment groups (^, *p* < 0.05; Mantel log-rank test). Treatment with Ad-Flt3L, or Ad-TK alone significantly improved survival when compared to saline (*, *p* < 0.05; Mantel log-rank test). (B) 7 d after treatment, tumor-bearing animals were humanely killed and HMGB1 release was assessed in the serum. Treatment of GL261 tumor-bearing animals with Ad-Flt3L and Ad-TK (+GCV) significantly increased the levels of HMGB1 in the serum when compared to tumor-bearing animals treated with saline. *, p < 0.05 versus saline (Mann-Whitney *U*-test). (C) B16-F10 cells were implanted into the striatum of C57/Bl6 mice. 17 d later animals were treated with Ad-Flt3L, Ad-TK (+GCV), Ad-Flt3L plus Ad-TK (+GCV), or saline. Treatment with Ad-Flt3L and Ad-TK significantly improved survival when compared to saline (*, *p* < 0.05; Mantel log-rank test), Ad-Flt3L, or Ad-TK alone treatment groups (^, *p* < 0.05; Mantel log-rank test). Treatment with Ad-Flt3L, or Ad-TK alone, significantly improved survival when compared to saline (*, *p* < 0.05; Mantel log-rank test). (D) 7 d after treatment, tumor-bearing animals were humanely killed and HMGB1 release was assessed in the serum. Treatment of B16-F10 tumor-bearing animals with Ad-Flt3L and Ad-TK (+GCV) significantly increased the levels of HMGB1 in the serum when compared to tumor-bearing animals treated with saline. *, p < 0.05 versus saline (Mann-Whitney *U*-test).

We next investigated the therapeutic efficacy of Ad-Flt3L and Ad-TK (+GCV) in vivo in mice bearing intracranial tumors derived from GL261 or B16-F10 cells and whether HMGB1 was released into their serum following treatment. We observed that intratumoral delivery of Ad-Flt3L and Ad-TK (+GCV) elicited long-term survival (>60 d) in 60% of WT mice bearing GL261 (*, *p* < 0.05 versus saline; Figure 10A) and in 40% of mice bearing B16-F10 tumors (*, *p* < 0.05 versus saline; MSR 1.57; Figure 10C). Ad-Flt3L or Ad-TK failed to elicit long-term survival when administered individually. Levels of HMGB1 were increased in the serum of mice bearing either GL261 (*, *p* < 0.05; η^2^ = 0.67; Figure 10B) or B16-F10 tumors (*, *p* < 0.05; η^2^ = 0.54; Figure 10D) 7 d after treatment with Flt3L and TK. These data demonstrating therapeutic efficacy of intratumoral administration of Ad-Flt3L and Ad-TK in tumors derived from two other cell lines suggest that this combined immunotherapy could be effective against a wide range of primary and metastatic brain tumors. Also, increased levels of HMGB1 in the serum of mice bearing GL261 or B16-F10 tumors after treatment with Ad-Flt3L and Ad-TK indicate that HMGB1 release is not limited to tumor derived from GL26 cells, and in fact, is released from tumors originating from various other cell types.

## Discussion

This paper elucidates the mechanisms that mediate an antibrain tumor immune response directly from within the tumor microenvironment. Utilizing syngeneic brain tumor models we demonstrate that it is possible to stimulate a systemic antitumor immune response by modifying the brain tumor microenvironment through the recruitment of DCs to the tumor milieu in combination with tumor cell killing. Further, we demonstrate an essential link that has so far been difficult to determine. Namely, it has been known for some time that stimulation of the innate immune system is necessary to achieve activation of the systemic adaptive immune response. However, how this is achieved in tumors, in the absence of exogenous activators of TLR signaling has remained elusive. Clinically, tumor immunization paradigms have attempted to provide innate immune stimulation through the administration of vaccines with known TLR activators, such as CpG [64,65].

**Figure 11:**
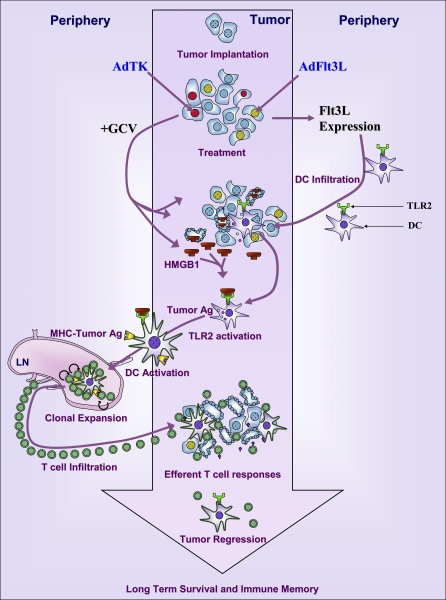
Model Illustrating the Role of TLR2 and HMGB1 in Initiating T Cell-Dependent Brain Tumor Regression by Flt3L and TK Treatment of tumor cells with TK + GCV releases endogenous TLR2 ligands including HMGB1. DC infiltration into tumors and subsequent activation require TLR2 signalling. DC phagocytose tumor antigen and migrate to the dLN where they stimulate T cell clonal expansion resulting in T cell-dependent brain tumor regression and long-term immunological memory.

Herein, we demonstrate that treatment of brain tumors with Ad-TK (+GCV) and Ad-Flt3L injected directly into the brain tumor microenvironment stimulates a systemic adaptive antiglioma immune response. Furthermore, we show that this treatment is strictly dependent on the activation of TLR signaling on bone-marrow DCs that infiltrate the tumor. We went further to identify glioma-derived HMGB1 as the endogenous TLR2 ligand, whose signaling is necessary to elicit systemic adaptive immune-mediated GBM regression and long-term immunological memory in an intracranial glioma model (Figure 11) [51,66].

Injured tissue can trigger inflammatory and auto-immune responses owing to the release of endogenous molecules that activate signaling cascades that mediate DC activation and T cell clonal expansion [67]. Several injury-associated molecules have been recently identified that mediate their effects by signaling through TLRs. For example, TLR2 signaling can be stimulated in response to host-derived molecules including gp96 [57,68], HMGB1 [69], low molecular weight hyaluronan fragments [55], soluble CD14 [29,70], and possibly larger hydrophobic molecular complexes (Hyppo's) [71]_._

Many of these endogenous ligands, such as HMGB1 [72], CD14 [70], and HSP70 [73], are expressed by brain tumor cells. Here we show that dying glioma cells released HMGB1, as a result of infection and killing with Ad-TK (+GCV); HMGB1 in turn stimulated TLR2-dependent NFκB signaling and DC activation. Importantly, we never observed any evidence for brain autoimmunity in these studies, or in our prior work [8,74–76], nor is it observed clinically in patients surviving a stroke, or following brain surgery [2]. Further, T cell-dependent tumor regression failed in vivo in TLR2 knockout mice, or mice where HMGB1 is either depleted or functionally inactivated. This strongly suggests that HMGB1 is the main, if not unique, endogenous activator of TLR2 signaling and necessary innate immune activation.

We used two different inhibitors of HMGB1 to block the activity of the putative endogenous TLR2 ligand during the combined immunotherapy-induced tumor regression. Glycyrrhizin binds to both box domains within HMGB1 [40,63] and thus prevents subsequent HMGB1 signaling; specific polyclonal blocking antibodies to HMGB1 [39,77–80] inactivate HMGB1 by binding to it, and reducing its bioavailability. Both pathways of HMGB1 antagonism completely abrogated the therapeutic efficacy of Ad-Flt3L plus Ad-TK. Glycyrrhizin binding to HMGB1 effectively inhibits its chemoattractant properties [63]. Glycyrrhizin has also been used to inhibit HMGB1-mediated liver disease in a model of viral hepatitis [40]. Other investigators have used different polycolonal antibodies raised against small peptides within Box A [39,77] or Box B [78–80] of HMGB1 to successfully inhibit HMGB1 activity in animal models of collagen-induced arthritis, sepsis, liver ischemia reperfusion injury, and intestinal barrier dysfunction following hemorrhagic shock. Whether additional proteins released from the tumor are able to stimulate other TLR pathways has not been determined. Importantly for our experimental paradigm, however, blocking HMGB1 alone was sufficient to completely abrogate the efficacy of the antiglioma treatment.

Our results demonstrate that glioma-derived HMGB1 released from dying cells is necessary for the clonal expansion of CD8^+^ T cells specific for glioma antigens including the Trp2_180–188_ peptide (H-2K^b^), and that the effects of HMGB1 are mediated through TLR2 signaling on tumor-infiltrating DCs. Importantly, HMGB1 released from dying GBM cells after treatment with TK (+GCV), on its own, was insufficient to induce tumor regression in our model. This result is shown by the failure of Ad-TK (+GCV) alone to eliminate brain tumors. Only the combined treatment of tumors with Ad-Flt3L and Ad-TK (+GCV) overcame immune ignorance to tumor antigens located within the brain. This indicates that expression of Flt3L is necessary to induce the migration and differentiation of DCs within the tumor. By expressing Flt3L directly within the brain tumor we achieved infiltration of immune cells directly into the brain tumor microenvironment, and stimulation of a systemic antitumor immune response. We have previously shown that Flt3L recruits DCs into the naïve rat brain [7]. However, recruitment of immune cells on its own also fails to eliminate the brain tumors [74]. Thus, both Flt3L-induced recruitment of immune cells to the brain and TK-dependent release of the TLR2 agonist HMGB1 are necessary for tumor elimination; together, they are sufficient to induce the regression of a large intracranial glioma and melanoma tumors.

It is likely that HMGB1 released into the serum as a result of tumor cell death mediates TLR2 signaling on BMDCs, facilitating their subsequent activation within the tumor [81]. Our data indicate that DC do not infiltrate into the tumor mass of TLR2^−/−^ mice after treatment with Flt3L and TK (+GCV). The absence of TLR2 signaling on brain-infiltrating immune cells abrogates tumor elimination. Thus, Flt3L is necessary to recruit the appropriate immune cell types to the brain tumor microenvironment, but TLR2 signaling must then be activated on these cells by HMGB1 released from dying tumor cells. Our data demonstrate that HMGB1-mediated TLR2 signaling links the effects of Flt3L on the recruitment of immune cells to the brain tumor microenvironment to their capacity to induce a systemic antitumor immune response.

Our data show that TLR2 signaling in response to endogenous HMGB1 ligand released from dying tumor cells needs to take place on BMDCs. Although our experiments suggest that HMGB1 signaling occurs mainly through TLR2 activation, we cannot discard that other endogenous ligands may be released that may act on TLR4, although our data in TLR4^−/−^ mice do not lend strong support for the role of TLR4 signaling in our model.

Our results also demonstrate long-term survival and increased levels of circulating HMGB1 in the serum of Ad-Flt3L + Ad-TK (+GCV) treated animals bearing intracranial melanomas. These data broaden the potential therapeutic use of Ad-Flt3L + Ad-TK for the treatment of other solid tumors, and suggest that the same molecular mechanism described initially could also be responsible for the elimination of metastatic brain tumors. While the detailed experiments to elucidate the role of HMGB1 and TLR2 signaling have only been performed using the GL26 cells in C57Bl/6 mice, we believe that the general effectiveness of the Ad-Flt3L and Ad-TK therapeutic approach is likely to be mediated by the same general underlying mechanism in both primary and metastatic brain tumors [8,75,76]. The endogenous activation of TLR signaling explains how activation of the innate immune response can be achieved through tumor-derived ligands during the induction of therapeutically effective and selective antibrain tumor immune responses.

In conclusion, the results reported provide compelling evidence for the role played by HMGB1 in mediating the efficacy of antiglioma therapeutic regimes that are based on tumor cell killing strategies, such as the HSV1-TK (+GCV) conditional cytotoxic approach described herein and currently in Phase III clinical trials [82], or other currently employed chemotherapeutic (e.g., temozolomide), and radiotherapy regimes. A recent case study suggesting a potential synergistic effect between temozolomide and an immunotherapy involving a tumor vaccination strategy [83] further supports our hypothesis that cancer immunotherapies coupled with effective cell killing modalities may be necessary to achieve therapeutically relevant antitumor efficacy. Moreover, results reported in a Phase I DC vaccine trial for malignant astrocytoma, highlights the role of chemotherapy in enhancing DC mediated antitumor immune responses [84].

If endogenous TLR2 signaling is necessary for the activation of immune responses against tumor antigens by the immune system, one would expect to see an increased incidence of cancer correlating with mutations in TLR2. Genetic evidence recently emerged supporting this hypothesis. SNPs in TLR2 have been correlated with increased incidence of lymphoma [23] and colorectal cancer [22]. Moreover, several SNP in the TLR6-1-10 gene cluster (TLR2 heterodimeric partners) are associated with the progression of prostate cancer [24,25]. Thus, the findings reported underscore the role of tumor-derived TLR2 ligands in mediating cancer regression in response to anticancer therapeutics impacting the implementation of brain cancer immunotherapies in human patients. We believe that the molecular and cellular mechanisms uncovered herein provide compelling evidence for the use of immunotherapies in combination with tumor killing strategies, such as radiotherapy, chemotherapy, or gene therapy for the effective treatment and eradication of primary brain gliomas and metastatic brain tumors. Based on the data reported here, Ad-TK and Ad-Flt3L have now been chosen for further downstream process development, manufactured under good clinical manufacturing processes, and will be tested in early phase clinical trials for the treatment of resectable, recurrent glioma in the near future.

## Supporting Information

Alternative Language Abstract S1 Chinese Translation of the Abstract by Naiyou Liu (23 KB DOC)

Alternative Language Abstract S2 Portuguese Translation of the Abstract by Marianela Candolfi (40 KB DOC)

Alternative Language Abstract S3 Spanish Translation of the Abstract by Marianela Candolfi (36 KB DOC)

Alternative Language Abstract S4 German Translation of the Abstract by Kathrin Michelsen (36 KB DOC)

Alternative Language Abstract S5 Japanese Translation of the Abstract by Yohei Mineharu (37 KB DOC)

Alternative Language Abstract S6 Russian Translation of the Abstract by Alexander V. Ljubimov (27 KB DOC)

Alternative Language Abstract S7 French Translation of the Abstract by Séverine Rémy (28 KB DOC)

Figure S1 GL26 Cells Do Not Contain Y Chromosomal DNA Genomic DNA was purified from GL26 tumor cells or from male C57BL/6 mouse lymphocytes as a positive control using DNeasy (Qiagen). PCR was used to amplify the male chromosome specific SRY sequence from 1 ng genomic DNA. GAPDH was also amplified as a control for DNA loading. GL26 cells do not contain Y chromosomal DNA and all animals used throughout this study were female to limit the recognition of proteins expressed at different levels in male mice.
(1.3 MB TIF)

Figure S2 Generating GFP Chimeric Mice (A) WT mice were irradiated and bone marrow from GFP^+/+^ mice was transferred into the irradiated WT mice to generate mice with GFP^+/+^ bone marrow-derived immune cells and WT microglia.
(B) GFP^+/+^ mice were irradiated and bone marrow from WT mice was transferred into the irradiated GFP^+/+^ mice to generate chimeric mice with WT bone marrow-derived immune cells and GFP^+/+^ microglia. As controls, WT bone marrow was transferred into WT mice (GFP bone marrow-derived leukocytes and microglia) and GFP^+/+^ bone marrow was transferred into GFP^+/+^ mice (GFP^+^ bone marrow-derived leukocytes and microglia).
(1.7 MB TIF)

Figure S3 Development and Characterization of Polycolonal HMGB1 Antibody (A) Hopp-Woods and Kyte-Doolittle hydrophobicity plots of mouse HMGB1 were generated using BioToolKit software. HMGB1-depleting antibodies were raised in rabbits against the N-terminal sequence MGKGDPKKPRG.
(B) Anti-HMGB1 antibodies raised in rabbit are immunoreactive for HMGB1 (∼30 kDa) by western blot. Western blot on untreated GL26 cell lysates using Protein G affinity purified, rabbit polyclonal antibody raised against the N terminus of mouse HMGB1. HMGB1 protein migrated at ∼30 kDa.
(1.5 MB TIF)

Figure S4 Treatment of GL26 Brain Tumors with Flt3L and TK Induces T Cell Activation in dLN We assessed the activation status of T cells in tumor-bearing wt C57BL/6 mice (black) or *TLR2^−/−^* mice (red) treated with saline (S), Ad0 (0), AdFlt3L (F), AdTK (T), or AdFlt3L + AdTK (F/T) 17 d after tumor implantation. Animals were killed 7 d later and T cells were isolated from the dLN. Proliferation of CD4+ T cells (a) and CD8+ T cells (b) was determined by limited dilution of CFSE using flow cytometry (*, *p* < 0.05 versus saline; Kruskal-Wallis followed by Dunn's test).
(937 KB TIF)

Figure S5 TLR2 Activation by Tumor Extracts (A) To determine whether components of GL26 cells could stimulate TLR2 or TLR4 signaling, we cotransfected HEK-293 cells with pGL3-NFκB (expressing FFLuc) and pTK-rLuciferase (expressing rLuc) either alone (black, mock), or with pCMV-TLR2 (red, pTLR2), or with pCMV-TLR4 and pEF1α-MD2 (magenta, pTLR4). After 24 h, cells were incubated either with GL26 cell extract, PAM3CSK4 (specific TLR2 ligand), Escherichia coli K12 LPS (specific TLR4 ligand), or unstimulated control. Renilla and Firefly Luciferase expression was measured using Dual luciferase kit. NFκB activity was calculated based on normalized FFLuc reporter activity (FFLuc/rLuc). *, *p* < 0.05 versus unstimulated controls (Kruskal-Wallis followed by Dunn's test).
(B) We next determined whether extracts of other tumor cell lines might also stimulate TLR2 activity. HEK-293 cells were cotransfected with pGL3-NFκB and pRL-TK either alone (black, mock) or with pCMV-TLR2 (green, TLR2). After 24 h cells were incubated with tumor cell extract from GL26 (mouse glioma), LLc1 (mouse lung carcinoma), CNS1 (rat glioma), or with DSL6A (rat pancreatic carcinoma). Inset: cells were incubated with 100 ng/ml PAM3 (PAM3CSK4) as a positive control. Normalized FFluc reporter activity (FFLuc/rLuc) was used to determine NFκB activity (*, *p* < 0.05 versus corresponding mock; Mann-Whitney *U*-test).
(2 MB TIF)

Figure S6 AdTK and GCV Induce GL26 Cell Death In Vitro GL26 cells were mock infected or infected with Ad0, AdTK, or with AdTK, and media was supplemented with GCV. (A) 3 d later cells were stained with Annexin-V FITC and cell death was assessed using flow cytometry (*, *p* < 0.05 versus saline; Kruskal-Wallis followed by Dunn's test).
(B) Dead cells are evident using light microscopy in GL26 cell cultures treated with Ad-TK + GCV.
(C) Genomic DNA was isolated from 1 × 10^7^ GL26 cells infected with Ad-0, Ad-TK, or Ad-TK + GCV or mock infected cells 72 h after the addition of GCV and run on a 2% agarose gel. Only treatment with AdTK + GCV resulted in a DNA fragmentation characteristic of apoptotic cells.
(5.3 MB TIF)

## Abbreviations

Ad: adenoviral vector
BMDC: bone marrow-derived dendritic cell
DC: dendritic cell
dLN: draining lymph node
Flt3L: Fms-like tyrosine kinase 3 ligand
GCV: gancyclovir
GFP: green fluorescent protein
GBM: glioblastoma multiforme
HMGB1: high-mobility-group box 1
IL6: interleukin 6
MACS: magnetic activated cell sorter
mDC: myeloid DC
MSR: median survival ratio
TK: thymidine kinase
TLR: Toll-like receptor
TNFα: tumor necrosis factor α
