# Supplementary material for: HMGB1 Mediates Endogenous TLR2 Activation and Brain Tumor Regression
Source: PLoS Med. 2009 Jan 13;6(1):e1000010. doi: 10.1371/journal.pmed.1000010 (PMC2621261; doi:10.1371/journal.pmed.1000010)
Supplement: Supplementary file 1 [file pmed.1000010.sd001.doc]

**Chinese translation of the abstract for PLoS Medicine (By Naiyou Liu)**

Title (题目): 高迁移率族蛋白1（HMGB1）调节内源性Toll样受体2 (TLR2)信号通路激活和脑瘤衰退

James F. Curtin^1,4^, Naiyou Liu^1,4^, Marianela Candolfi^1,4^, Weidong Xiong ^1,4^, Hikmat Assi ^1,4^, Kader Yagiz^1,4^, Matthew R. Edwards^1^, Kathrin S. Michelsen^2^, Kurt M. Kroeger^1,4^, Chunyan Liu^1^, A.K.M. Ghulam Muhammad^1,4^, Mary C. Clark^1,4^, Moshe Arditi^3^, Begonya Comin-Anduix^5^, Antoni Ribas^5,7^, Pedro R. Lowenstein^1,4,5,6,7^, Maria G. Castro^1,4,5,6,7^

^1^Board of Governors’ Gene Therapeutics Research Institute and Department of Medicine, ^2^ Inflammatory Bowel Disease Center and Immunobiology Research Institute, ^3^Division of Pediatric Infectious Diseases, Cedars Sinai Medical Center, 8700 Beverly Blvd, Los Angeles, CA 90048, USA, ^4^Department of Molecular and Medical Pharmacology, ^5^Department of Surgery, ^6^The Brain Research Institute, and ^7^Jonsson Comprehensive Cancer Center, David Geffen School of Medicine, UCLA, Los Angeles, CA, 90095, USA.

**摘要**

**背景**

多形性胶质母细胞瘤（GBM）是恶性程度最高的原发性脑肿瘤，五年生存率仅为5 ％ 。由于大脑免疫赦免、肿瘤免疫逃逸以及中枢神经系统中缺乏树突状细胞（DCs），临床上促使脑肿瘤患者产生相关的抗GBM免疫反应的尝试并没有取得显著的成功。在此我们研究发现了一全新的可以有效激活抗GBM免疫反应的途径，该途径通过凋亡的肿瘤细胞所释放的一种报警信号蛋白--高迁移率族蛋白1 （HMGB1）所介导 ，此蛋白作为Toll样受体2（TLR2）信号通路的一个内源性配体，通过使骨髓衍生的树突状细胞浸润到GBM区而起作用。

**方法与结果**

通过使用免疫疗法和细胞毒性依赖的联合治疗方法，即利用腺病毒载体（Ad）携带表达Fms样酪氨酸激酶3配基（Flt3L）和胸苷激酶（TK）并将其导入瘤内，我们证明 CD4 ^+^和CD8 ^+^ T细胞是肿瘤衰退和免疫记忆所必须的。我们发现此治疗使侵润到肿瘤的骨髓衍生的髓样树突状细胞细胞（mDCs）大大增多；而且mDCs到GBM的侵润，抗肿瘤T细胞的克隆化扩增，以及有效的抗GBM免疫反应的诱导均依赖于TLR2。我们接着鉴定出HMGB1是负责肿瘤浸润性mDCs密度和激活的TLR2信号的内源性配体；用Ad-TK（+丙氧鸟苷）处理后，该蛋白从凋亡中的肿瘤细胞释放出来，在用Ad-Flt3L/Ad-TK （+丙氧鸟苷）治疗的荷瘤小鼠的血清中也可以检测到HMGB1蛋白水平的提高。 研究表明，TLR2信号能够被Ad-TK（+丙氧鸟苷）处理过的GBM细胞的上清特异激活，同时却被甘草甜素（HMGB1的一种特异抑制剂）或者HMGB1蛋白的抗体所阻断。用射线或者替莫唑胺处理过的黑色素瘤细胞、小细胞肺癌和神经胶质瘤细胞中也释放出HMGB1蛋白，而对经过Ad-Flt3L和Ad-TK（+丙氧鸟苷）治疗的荷瘤小鼠施加甘草甜素或HMGB1蛋白抗体却使得治疗效果全部消失，这更加突出了HMGB1介导的TLR2信号在肿瘤衰退中所发挥的关键作用。Ad-Flt3L和Ad-TK（+丙氧鸟苷）的治疗疗效在第二种胶质瘤模型和颅内黑色素瘤模型中进一步得到证明，同时伴随着外周血HMGB1蛋白水平的增加。

**结论**

我们的数据在分子和细胞机制方面提供了证据，它表明临床上实施抗脑肿瘤免疫治疗与其他杀死肿瘤的疗法相结合的联合治疗方案以产生有效的抗肿瘤免疫反应是完全可行的，这一理论基础也必将对神经肿瘤的临床实践产生深远的影响。
