# Supplementary material for: HMGB1 Mediates Endogenous TLR2 Activation and Brain Tumor Regression
Source: PLoS Med. 2009 Jan 13;6(1):e1000010. doi: 10.1371/journal.pmed.1000010 (PMC2621261; doi:10.1371/journal.pmed.1000010)
Supplement: Supplementary file 2 [file pmed.1000010.sd002.doc]

**HMGB1 media a ativação endógena de TLR2 e a regressão de tumores cerebrais**

James F. Curtin^1,4^, Naiyou Liu^1,4^, Marianela Candolfi^1,4^, Weidong Xiong ^1,4^, Hikmat Assi ^1,4^, Kader Yagiz^1,4^, Matthew R. Edwards^1^, Kathrin S. Michelsen^2^, Kurt M. Kroeger^1,4^, Chunyan Liu^1^, A.K.M. Ghulam Muhammad^1,4^, Mary C. Clark^1,4^, Moshe Arditi^3^, Begonya Comin-Anduix^5^, Antoni Ribas^5,7^, Pedro R. Lowenstein^1,4,5,6,7^, Maria G. Castro^1,4,5,6,7^

^1^Board of Governors’ Gene Therapeutics Research Institute and Department of Medicine, ^2^ Inflammatory Bowel Disease Center and Immunobiology Research Institute, ^3^Division of Pediatric Infectious Diseases, Cedars Sinai Medical Center, 8700 Beverly Blvd, Los Angeles, CA 90048, USA, ^4^Department of Molecular and Medical Pharmacology, ^5^Department of Surgery, ^6^The Brain Research Institute, and ^7^Jonsson Comprehensive Cancer Center, David Geffen School of Medicine, UCLA, Los Angeles, CA, 90095, USA.

**Resumo**

**Antecedentes**

O glioblastoma multiforme (GBM) é o tumor primário mais agressivo do sistema nervoso central (SNC), tendo uma taxa de sobrevivência de 5 anos de 5%. As tentativas de induzir respostas imunes contra o GBM que sejam clinicamente relevantes tem tido êxito limitado em pacientes com este tipo de câncer. Isto é devido a natureza imunoprivilegiada do SNC, a evasão tumoral à resposta imune e a escassez de células dendríticas no SNC. Aqui revelamos uma nova via para a ativação de uma resposta imune eficaz contra o GBM, mediada pela liberação da proteína *high-mobility-group box 1* (HMGB1), uma alarmina liberada pelas células tumorais durante a morte celular, a qual atua como um ligante endógeno para a sinalização através do receptor TLR2 nas células dendríticas originadas na medula óssea que infiltram o GBM.

**Métodos e Resultados**

Usando uma combinação de imunoterapia e citotoxicidade condicional que consiste em vetores adenovirais (Ad) que expressam *Fms-like Tyrosine Kinase 3 ligand* (Flt3L) e timidina quinase (TK) administrados na massa tumoral, demonstramos que as células T CD4^+^ e CD8^+^ são requeridas para a regressão do tumor e a indução da memória imunológica. Observou-se um aumento do número de células dendríticas mielóides (mDCs), de origem medular, infiltrando o tumor em resposta à terapia. Tanto a infiltração de mDCs no GBM, quanto a expansão clonal de células T anti-tumorais e a indução de uma resposta imune anti-tumoral efetiva foram dependentes de TLR2. Então, procedemos à identificação do ligante endogeno responsável pela sinalização de TLR2 nas mDCs que infiltram o tumor. Assim demonstramos que a proteína HMGB1 é liberada pelas células tumorais durante a morte celular induzida pelo tratamento com Ad-TK (+GCV). Também detectou-se um aumento dos níveis séricos de HMGB1 nos camundongos portadores de tumor tratados com Ad-Flt3L/Ad-TK (+GCV). A ativação específica de TLR2 foi induzida utilizando sobrenadante de células de GBM tratadas com Ad+TK (+GCV); este efeito foi bloqueado com glycyrrhizin (um inibidor específico de HMGB1) ou com anticorpos contra HMGB1. A proteína HMGB1 também foi liberada por células de melanoma, células de carcinoma pulmonar de células pequenas e por células de glioma tratadas com temozolamida ou irradiadas. A administração de glycyrrhizin ou de imunoglobulinas contra HMGB1 nos camundongos portadores de tumores tratados com Ad-Flt3L+AdTK bloqueou a eficácia terapêutica, ressaltando o papel crítico que exerce a sinalização de TLR2 induzida por HMGB1 na regressão tumoral. A eficácia terapêutica do tratamento com Ad-Flt3L+AdTK (+GCV) foi demonstrada num segundo modelo de glioma e num modelo de melanoma intracranial, com um aumento concomitante dos níveis circulantes de HMGB1.

**Conclusões**

Os mecanismos moleculares e celulares descritos neste trabalho avalizam a implementação clínica de imunoterapias anti-tumorais combinadas com a indução de morte de células tumorais para assim promover respostas imunes efetivas contra tumores do SNC, causando impacto na prática neuro-oncológica.
