# Supplementary material for: HMGB1 Mediates Endogenous TLR2 Activation and Brain Tumor Regression
Source: PLoS Med. 2009 Jan 13;6(1):e1000010. doi: 10.1371/journal.pmed.1000010 (PMC2621261; doi:10.1371/journal.pmed.1000010)
Supplement: Supplementary file 3 [file pmed.1000010.sd003.doc]

**HMGB1 media la activación endógena de TLR2 y la regresión de tumores cerebrales**

James F. Curtin^1,4^, Naiyou Liu^1,4^, Marianela Candolfi^1,4^, Weidong Xiong ^1,4^, Hikmat Assi ^1,4^, Kader Yagiz^1,4^, Matthew R. Edwards^1^, Kathrin S. Michelsen^2^, Kurt M. Kroeger^1,4^, Chunyan Liu^1^, A.K.M. Ghulam Muhammad^1,4^, Mary C. Clark^1,4^, Moshe Arditi^3^, Begonya Comin-Anduix^5^, Antoni Ribas^5,7^, Pedro R. Lowenstein^1,4,5,6,7^, Maria G. Castro^1,4,5,6,7^

^1^Board of Governors’ Gene Therapeutics Research Institute and Department of Medicine, ^2^ Inflammatory Bowel Disease Center and Immunobiology Research Institute, ^3^Division of Pediatric Infectious Diseases, Cedars Sinai Medical Center, 8700 Beverly Blvd, Los Angeles, CA 90048, USA, ^4^Department of Molecular and Medical Pharmacology, ^5^Department of Surgery, ^6^The Brain Research Institute, and ^7^Jonsson Comprehensive Cancer Center, David Geffen School of Medicine, UCLA, Los Angeles, CA, 90095, USA.

**Resumen**

**Antecedentes**

El glioblastoma multiforme (GBM) es el tumor primario más agresivo del sistema nervioso central (SNC), teniendo una tasa de supervivencia a 5 años del 5%. Los intentos de inducir respuestas inmunes contra el GBM que sean clínicamente relevantes han tenido éxito limitado en pacientes con estos tumores. Esto es debido a la condición de imunoprivilegio del SNC, a la evasión tumoral de la respuesta inmune y a la escasez de células dendríticas en el SNC. Aquí revelamos una nueva ruta para la activación de una respuesta inmune eficaz contra el GBM, mediada por la liberación de la proteína *high-mobility-group box 1* (HMGB1), una alarmina liberada por células tumorales durante la muerte celular, que actúa como un ligando endógeno para la señalización a través del receptor TLR2 en células dendríticas que infiltran el GBM, provenientes de la medula ósea.

**Métodos y Resultados**

Usando una combinación de inmunoterapia y citotoxicidad condicional que consiste en vectores adenovirales (Ad) que expresan *Fms-like Tyrosine Kinase 3 ligand* (Flt3L) y timidina quinasa (TK) administrados en la masa tumoral, demostramos que las células T CD4^+^ y CD8^+^ son requeridas para la regresión del tumor y la inducción de memoria inmunológica. Se observó un aumento en el número de células dendríticas mieloides (mDCs), de origen medular, infiltrando el tumor en respuesta a la terapia. Tanto la infiltración de mDCs en el GBM, como la expansión clonal de células T antitumorales y la inducción de una respuesta inmune antitumoral efectiva fueron dependientes de TLR2. Entonces, procedimos a identificar el ligando endógeno responsable por la señalización de TLR2 en las mDCs que infiltran el tumor. Demostramos que la proteína HMGB1 es liberada de las células tumorales durante la muerte celular inducida por el tratamiento con Ad-TK (+GCV). También se detectó un aumento en los niveles séricos de HMGB1 en los ratones portadores de tumor tratados con Ad-Flt3L/Ad-TK (+GCV). La activación especifica de TLR2 fue inducida utilizando el sobrenadante de células de GBM tratadas con Ad+TK (+GCV); este efecto fue bloqueado con glycyrrhizin (un inhibidor especifico de HMGB1) o con anticuerpos contra HMGB1. La proteína HMGB1 fue también liberada por células de melanoma, células de carcinoma de pulmón de células pequeñas y por células de glioma tratadas con temozolamida o irradiadas. La administración de glycyrrhizin o de inmunoglobulinas contra HMGB1 a los ratones portadores de tumores tratados con Ad-Flt3L+AdTK bloqueó la eficacia terapéutica, resaltando el papel crítico que juega la señalización de TLR2 inducida por HMGB1 en la regresión tumoral. La eficacia terapéutica del tratamiento con Ad-Flt3L+AdTK (+GCV) fue demostrada en un segundo modelo de glioma y en un modelo de melanoma intracraneal, acompañada de un aumento concomitante en los niveles circulantes de HMGB1.

**Conclusiones**

Los mecanismos moleculares y celulares descriptos en este trabajo avalan la implementación clínica de inmunoterapias antitumorales combinadas con la inducción de muerte de células tumorales para promover respuestas inmunes efectivas contra tumores del SNC, impactando así la práctica neuro-oncológica.
