# Supplementary material for: HMGB1 Mediates Endogenous TLR2 Activation and Brain Tumor Regression
Source: PLoS Med. 2009 Jan 13;6(1):e1000010. doi: 10.1371/journal.pmed.1000010 (PMC2621261; doi:10.1371/journal.pmed.1000010)
Supplement: Supplementary file 4 [file pmed.1000010.sd004.doc]

**HMGB1 vermittelt endogene TLR2 Aktivierung und Gehirntumor Regression**

James F. Curtin^1,4^, Naiyou Liu^1,4^, Marianela Candolfi^1,4^, Weidong Xiong ^1,4^, Hikmat Assi ^1,4^, Kader Yagiz^1,4^, Matthew R. Edwards^1^, Kathrin S. Michelsen^2^, Kurt M. Kroeger^1,4^, Chunyan Liu^1^, A.K.M. Ghulam Muhammad^1,4^, Mary C. Clark^1,4^, Moshe Arditi^3^, Begonya Comin-Anduix^5^, Antoni Ribas^5,7^, Pedro R. Lowenstein^1,4,5,6,7^, Maria G. Castro^1,4,5,6,7^

^1^Board of Governors’ Gene Therapeutics Research Institute and Department of Medicine, ^2^ Inflammatory Bowel Disease Center and Immunobiology Research Institute, ^3^Division of Pediatric Infectious Diseases, Cedars Sinai Medical Center, 8700 Beverly Blvd, Los Angeles, CA 90048, USA, ^4^Department of Molecular and Medical Pharmacology, ^5^Department of Surgery, ^6^The Brain Research Institute, and ^7^Jonsson Comprehensive Cancer Center, David Geffen School of Medicine, UCLA, Los Angeles, CA, 90095, USA.

**Zusammenfassung**

**Hintergrund**

Glioblastome multiforme (GBM) ist der aggressivste, primäre Gehirntumor mit einer 5-Jahres Überlebensrate von 5 %. Die bisherigen Versuche, eine klinisch relevante Immunantwort gegen GBM in Patienten mit diesem Gehirntumor auszulösen, hatten nur begrenzte Erfolge. Dies ist auf das Immunprivileg des Gehirns, eine Umgehung des Immunsystems durch Tumorzellen und ein Fehlen von Dendritischen Zellen (DCs) im Zentralen Nervensystem, zurückzuführen. Wir haben hier einen neuartigen Mechanismus für die Aktivierung einer effektiven Immunantwort gegen GBM entdeckt: die Freisetzung von high-mobility-group box 1 (HMGB1), ein Alarm Protein, welches von sterbenden Tumorzellen freigesetzt wird. HMGB1 ist ein endogener Ligand für Toll-like Receptor 2 (TLR2), welcher auf Knochenmarks-abstammenden, GBM-infiltrierenden DCs exprimiert wird.

**Methoden und Ergebnisse**

Wir verwendeten eine kombinierte Therapie von Immuntherapie und konditionell, zytotoxischer Therapie indem wir adenovirale Vektoren, die Fsm-like Tyrosin Kinase 3 Liganden (Flt3L) und Thymidin Kinase (TK) exprimieren, direkt in den Tumor injizierten. Wir demonstrierten, dass CD4+ und CD8+ T Zellen für Tumorregression und immunologisches Gedächtnis notwendig sind. Wir beobachteten eine erhöhte Anzahl von Knochenmarks-abstammenden, Tumor-Infiltrierenden myeloiden DCs (mDCs) als Reaktion auf die Therapie. Die Infiltration von mDCs in GBM, klonale Expansion von Tumor-spezifischen T Zellen und die Induktion einer effektiven Immunantwort gegen GBM sind abhängig von TLR2. Als nächstes wollten wir den endogenen Liganden verantwortlich für TLR2 Signaltransduktion in Tumor-infiltrierenden DCs identifizieren. Wir demonstrierten, dass HMGB1 als Reaktion auf Ad-TK (+GCV) Behandlung von sterbenden Tumorzellen freigesetzt wird. Erhöhte Serumkonzentrationen vom HMGB1 konnten auch in GBM-erkrankten Mäusen, welche mit Ad-Flt3L/Ad-TK (+GCV) behandelt wurden, nachgewiesen werden. TLR2 Signaltransduktion wurde aktiviert durch Zellkulturüberstände von Ad-TK (+GCV) behandelten GBM Zellen, und TLR2 Signaltransduktion konnte durch Glycyrrhizin (einem spezifischen HMGB1 Inhibitor) oder Antikörpern gegen HMGB1 gehemmt werden. HMGB1 wurde auch von Melanoma, Lungenkarzimoa und Glioma Zellen nach Bestrahlung oder Temezolomide Behandlung freigesetzt. Gabe von entweder Glycyrrhizin oder Antikörpern gegen HMGB1 an GBM-erkrankten und Ad-Flt3L/Ad-TK-behandelten Mäusen hob die Effizienz der Therapie auf. Dies zeigt, dass HMGB1-vermittelte TLR2 Signaltransduktion eine kritische Rolle bei der Tumorregression spielt. Die therapeutische Wirksamkeit von Ad-Flt3L und Ad-TK (+GCV) Behandlung wurde auch in einem zweiten Glioma Modell und einem intracranialem Melanoma Modell bestätigt. In beiden Modellen waren die Serumkonzentrationen von HMGB1 erhöht.

**Schlussfolgerungen**

Unsere Daten liefern Beweise für molekulare und zelluläre Mechanismen, welche das Grundprinzip für die klinische Anwendung von einer Kombination aus Immuntherapie gegen Gehirntumore und Tumorabtötung unterstützen. Die Kombinationstherapie löst eine effektive Immunantwort gegen Tumorzellen aus und wird somit Bedeutung für die klinische, neuroonkologische Behandlung haben.
