# Supplementary material for: HMGB1 Mediates Endogenous TLR2 Activation and Brain Tumor Regression
Source: PLoS Med. 2009 Jan 13;6(1):e1000010. doi: 10.1371/journal.pmed.1000010 (PMC2621261; doi:10.1371/journal.pmed.1000010)
Supplement: Supplementary file 5 [file pmed.1000010.sd005.doc]

**HMGB1は内因性リガンドとしてTLR2を活性化し、脳腫瘍を退縮させる**

James F. Curtin^1,4^, Naiyou Liu^1,4^, Marianela Candolfi^1,4^, Weidong Xiong ^1,4^, Hikmat Assi ^1,4^, Kader Yagiz^1,4^, Matthew R. Edwards^1^, Kathrin S. Michelsen^2^, Kurt M. Kroeger^1,4^, Chunyan Liu^1^, A.K.M. Ghulam Muhammad^1,4^, Mary C. Clark^1,4^, Moshe Arditi^3^, Begonya Comin-Anduix^5^, Antoni Ribas^5,7^, Pedro R. Lowenstein^1,4,5,6,7^, Maria G. Castro^1,4,5,6,7^

^1^Board of Governors’ Gene Therapeutics Research Institute and Department of Medicine, ^2^ Inflammatory Bowel Disease Center and Immunobiology Research Institute, ^3^Division of Pediatric Infectious Diseases, Cedars Sinai Medical Center, 8700 Beverly Blvd, Los Angeles, CA 90048, USA, ^4^Department of Molecular and Medical Pharmacology, ^5^Department of Surgery, ^6^The Brain Research Institute, and ^7^Jonsson Comprehensive Cancer Center, David Geffen School of Medicine, UCLA, Los Angeles, CA, 90095, USA.

**論文要旨**

**背景**

多形膠芽腫（GBM）は、原発性脳腫瘍のうち最も成長が早く、5年生存率はわずか5%に過ぎない。GBM患者の抗腫瘍免疫応答を誘導して治療に結びつけようという試みがなされているが、臨床応用可能なレベルにまで達したものはごく一部しかない。免疫応答の誘導が困難な要因は、脳の免疫が特殊であること（immune privileged：免疫特権）、腫瘍が免疫機能を傷害すること、脳に樹状細胞が少ないことである。我々はこれらの点を克服し、抗GBM免疫応答を治療に有効なレベルにまで活性化することに成功した。そして、この免疫応答の活性化において、死滅腫瘍細胞（dying tumor cells）から放出されるalarminのひとつであるHMGB1(high-morbility-group box 1)が、GBMに侵入した骨髄由来樹状細胞上のToll like receptor 2（TLR2）の内因性リガンドとして働きシグナルを伝えるという新たな経路を発見した。

**方法と結果**

我々は、Fms-like Tyrosin Kinase 3 ligand (Flt3L)とTymidine Kinase (TK)を発現するアデノウイルスベクターを腫瘍に打ち込むという、免疫療法と細胞傷害療法を組み合わせた治療法を用いて、CD4+およびCD8+T細胞が腫瘍退縮と免疫記憶形成に必要であることを示した。そして、この治療法により、骨髄由来の樹状細胞が腫瘍内で増殖することが確認された。また、GBMへの樹状細胞の侵入、抗腫瘍T細胞のクローナルな増殖と有効な抗GBM免疫応答の誘導は、TLR2を介して行われることを示した。次にTLR2の内因性リガンドを検索したところ、HMGB1がAd-TK治療により死滅する細胞から放出されることがわかり、TLR2のリガンドである可能性が示唆された。血清中のHMGB1の上昇は、Ad-Flt3L/Ad-TK (+GCV)治療を行ったマウスでも認められた。TLR2シグナルは、Ad-TK (+GCV)を投与したGBM細胞の培養液上清により特異的に活性化され、HMGB1の特異的阻害剤であるグリチルリチンや抗HMGB1抗体で阻害された。なお、HMGB1は悪性黒色腫、肺小細胞癌、放射線治療やテモゾロミド治療を受けた神経膠細胞からも放出された。Ad-Flt3L/Ad-TK (+GCV)で治療した脳腫瘍モデルマウスに、グリチルリチンや抗HMGB1抗体を投与すると、治療効果が損なわれることが示され、HMGB1を介したTLR2シグナルが腫瘍退縮において決定的な役割を果たしていることが明らかとなった。Ad-Flt3L/Ad-TK (+GCV)治療は、神経膠腫再発モデル、頭蓋内悪性黒色腫において、HMGB1の血中濃度に依存して、効果が上がることも示された。

**結論**

我々の実験データは、細胞傷害療法に抗脳腫瘍免疫療法を組み合わせるという治療法が、効果的な抗腫瘍免疫応答を誘導するのに合理的であることを分子生物学的に証明しており、神経腫瘍の臨床現場において大変意義深い。

訳：峰晴陽平
