# Supplementary material for: HMGB1 Mediates Endogenous TLR2 Activation and Brain Tumor Regression
Source: PLoS Med. 2009 Jan 13;6(1):e1000010. doi: 10.1371/journal.pmed.1000010 (PMC2621261; doi:10.1371/journal.pmed.1000010)
Supplement: Supplementary file 6 [file pmed.1000010.sd006.doc]

**HMGB1 способствует активации эндогенного TLR2 и регрессии опухолей мозга.**

James F. Curtin^1,4^, Naiyou Liu^1,4^, Marianela Candolfi^1,4^, Weidong Xiong ^1,4^, Hikmat Assi ^1,4^, Kader Yagiz^1,4^, Matthew R. Edwards^1^, Kathrin S. Michelsen^2^, Kurt M. Kroeger^1,4^, Chunyan Liu^1^, A.K.M. Ghulam Muhammad^1,4^, Mary C. Clark^1,4^, Moshe Arditi^3^, Begonya Comin-Anduix^5^, Antoni Ribas^5,7^, Pedro R. Lowenstein^1,4,5,6,7^, Maria G. Castro^1,4,5,6,7^

^1^Board of Governors’ Gene Therapeutics Research Institute and Department of Medicine, ^2^ Inflammatory Bowel Disease Center and Immunobiology Research Institute, ^3^Division of Pediatric Infectious Diseases, Cedars Sinai Medical Center, 8700 Beverly Blvd, Los Angeles, CA 90048, USA, ^4^Department of Molecular and Medical Pharmacology, ^5^Department of Surgery, ^6^The Brain Research Institute, and ^7^Jonsson Comprehensive Cancer Center, David Geffen School of Medicine, UCLA, Los Angeles, CA, 90095, USA.

**Резюме**

**Введение**

Мультиформная глиобластома (МГБ) является наиболее агрессивной первичной опухолью мозга с пятилетней выживаемостью 5%. Попытки вызвать клинически значимый иммунный ответ на МГБ у больных с опухолями мозга имели ограниченный успех. Это связано с иммунной привилегированностью мозга, способностью опухоли ускользать от иммунологического надзора и незначительным количеством дендритных клеток (ДК) в центральной нервной системе. В данной работе мы обнаружили новый путь активации эффективного иммунного ответа на МГБ, осуществляемого с помощью выделения HMGB1 (бокс 1 высоко мобильной группы). Этот белок из группы аларминов высвобождается из отмирающих опухолевых клеток и действует в качестве эндогенного лиганда для проведения сигнала через рецептор TLR2 на ДК из костного мозга, инфильтрирующих МГБ.

**Методы и Результаты**

Была использована комбинация иммунотерапии и индуцибельной цитотоксичности, с применением аденовирусных векторов (Ад), экспрессирующих лиганд Fms-подобной тирозинкиназы 3 (Flt3L) или тимидинкиназу (ТК) и доставляемых в массу опухоли. Показано, что для регрессии опухолей и иммунологической памяти требуются CD4^+^ и CD8^+^ T клетки. Количество миелоидных ДК (мДК) из костного мозга, инфильтрирующих МГБ, увеличивалось в ответ на терапию. Инфильтрация мДК в ткань МГБ, клональная экспансия противоопухолевых T клеток и индукция эффективного иммунного ответа на МГБ зависели от TLR2. Авторы далее идентифицировали эндогенный лиганд, ответственный за активацию сигнала TLR2 на инфильтрирующих опухоль мДК. Было установлено, что HMGB1 высвобождался из отмирающих опухолевых клеток в ответ на цитотоксическую терапию с помощью Ад-ТК с ганцикловиром (ГЦВ). Повышенные уровни HMGB1 были также обнаружены в плазме мышей опухоленосителей, обработанных Ад-Flt3L/Ад-ТК+ГЦВ. Специфическая активация сигнала TLR2 была индуцирована супернатантами МГБ, обработанных Ад-ТК+ГЦВ. Этот эффект блокировался глициризином (специфическим ингибитором HMGB1) или антителами против HMGB1. HMGB1 также высвобождался из клеток меланомы, мелкоклеточного рака легкого и глиомы, обработанных радиацией или темозоламидом. Обработка мышей опухоленосителей после лечения Ад-Flt3L и Ад-ТК глициризином или антителами против HMGB1 приводила к потере терапевтической эффективности, что свидетельствовало об определяющей роли HMGB1-зависимого сигнала через TLR2 в индукции регрессии опухолей. Эффективность терапии с помощью Ад-Flt3L и Ад-ТК+ГЦВ была также продемонстрирована на второй модели глиомы и на модели внутричерепной меланомы, с сопутствующим увеличением уровней циркулирующего HMGB1.

Выводы

Полученные данные свидетельствуют о молекулярных и клеточных механизмах, согласующихся с концепцией о целесообразности клинического использования иммунотерпии против рака мозга в сочетании с цитотоксическими подходами. Такая терапия с целью индукции эффективного противоопухолевого иммунитета может оказать позитивное влияние на клиническую практику в нейроонкологии.
