# Supplementary material for: HMGB1 Mediates Endogenous TLR2 Activation and Brain Tumor Regression
Source: PLoS Med. 2009 Jan 13;6(1):e1000010. doi: 10.1371/journal.pmed.1000010 (PMC2621261; doi:10.1371/journal.pmed.1000010)
Supplement: Supplementary file 7 [file pmed.1000010.sd007.doc]

**HMGB1 endogène médie l’activation du TLR2 et la régression de tumeurs cérébrales.**

James F. Curtin^1,4^, Naiyou Liu^1,4^, Marianela Candolfi^1,4^, Weidong Xiong ^1,4^, Hikmat Assi ^1,4^, Kader Yagiz^1,4^, Matthew R. Edwards^1^, Kathrin S. Michelsen^2^, Kurt M. Kroeger^1,4^, Chunyan Liu^1^, A.K.M. Ghulam Muhammad^1,4^, Mary C. Clark^1,4^, Moshe Arditi^3^, Begonya Comin-Anduix^5^, Antoni Ribas^5,7^, Pedro R. Lowenstein^1,4,5,6,7^, Maria G. Castro^1,4,5,6,7^

^1^Board of Governors’ Gene Therapeutics Research Institute and Department of Medicine, ^2^ Inflammatory Bowel Disease Center and Immunobiology Research Institute, ^3^Division of Pediatric Infectious Diseases, Cedars Sinai Medical Center, 8700 Beverly Blvd, Los Angeles, CA 90048, USA, ^4^Department of Molecular and Medical Pharmacology, ^5^Department of Surgery, ^6^The Brain Research Institute, and ^7^Jonsson Comprehensive Cancer Center, David Geffen School of Medicine, UCLA, Los Angeles, CA, 90095, USA.

**Résumé**

**Contexte**

Le glioblastome multiforme (GBM) est la forme la plus agressive des tumeurs cérébrales portant le taux de survie à 5% à 5 ans. Les espoirs d’induire une réponse immunitaire anti-GBM efficace chez les patients atteints d’une tumeur cérébrale sont limités du fait du privilège immun du cerveau, de l’échappement tumorale à la réponse immunitaire et de la rareté des cellules dendritiques (CDs) au sein du système nerveux central. Dans cette étude, nous avons mis en évidence une nouvelle voie d’induction de la réponse immunitaire anti-GBM efficace, à travers la sécrétion de HMGB1 (high-mobility-group box 1), une protéine « de danger » passivement libérée par les cellules tumorales nécrotiques, qui se lie au TLR2 des CDs, dérivées de la moelle osseuse, infiltrants les GBM.

# Méthodes et Résultats

Par une approche reposant sur l’immunothérapie et la cytotoxicité, via l’injection au sein de la masse tumorale de vecteurs adénoviraux (Ad) codant pour le ligand Flt3 (Fms-like Tyrosine Kinase 3) et la Thymidine Kinase (TK), nous avons démontré que les cellules T CD4^+^ et CD8^+^ jouaient un rôle important dans la régression tumorale et la mémoire immunologique. Un nombre croissant de CDs myéloïdes (mCDs), dérivées de la moelle osseuse, infiltrantes la tumeur a été observé en réponse à la thérapie. L’infiltration des mCDs au sein des GBM, l’expansion clonale de cellules T anti-tumorales et l’induction d’une réponse immunitaire anti-GBM efficace sont TLR2-dépendantes. Nous avons cherché à identifier le ligand endogène qui se lie au TLR2 des mCDs infiltrants la tumeur. Nous avons démontré que HMGB1 était sécrété par les cellules tumorales nécrotiques, en réponse au traitement Ad-TK (+GCV). Des niveaux croissants de HMGB1 ont aussi été détectés dans le sérum de souris atteintes d’une tumeur et traitées par l’ Ad-Flt3L/Ad-TK (+GCV). L’activation spécifique du TLR2 a été induite par les surnageants de cellules GBM traitées par l’Ad-TK (+GCV) et bloquée par la glycyrrhizine (un inhibiteur spécifique d’HMGB1) ou par des anticorps anti- HMGB1. HMGB1est aussi sécrété par les cellules de mélanomes, les petites cellules de carcinomes pulmonaires et les cellules de gliomes, traitées par radiation ou par du témozolomide. L’administration soit de glycyrrhizine soit d’immunoglobulines anti- HMGB1 à des souris atteintes de tumeurs et traitées par l’ Ad-Flt3L et l’Ad-TK, abolit l’efficacité thérapeutique, soulignant le rôle critique de l’activation du TLR2 par HMGB1 dans la régression tumorale. L’efficacité thérapeutique du traitement Ad-Flt3L et Ad-TK (+GCV) a été démontrée dans un second modèle de gliomes et dans un modèle de mélanome intracrânien avec des augmentations concomitantes des niveaux circulants de HMGB1.

**Conclusions**

Cette étude a permis d’identifier les mécanismes cellulaires et moléculaires qui pourraient être utilisés en immunothérapie clinique des tumeurs cérébrales, pour augmenter efficacement le potentiel immunogénique des traitements anticancéreux conventionnels, et ainsi avoir des incidences sur les études neuro-oncologiques cliniques.
